# SINE transcription by RNA polymerase III is suppressed by histone methylation but not by DNA methylation

**DOI:** 10.1038/ncomms7569

**Published:** 2015-03-23

**Authors:** Dhaval Varshney, Jana Vavrova-Anderson, Andrew J. Oler, Victoria H. Cowling, Bradley R. Cairns, Robert J. White

**Affiliations:** 1 College of Medical, Veterinary and Life Sciences, University of Glasgow, Glasgow G12 8QQ, UK; 2 The MRC Protein Phosphorylation Unit, University of Dundee, Dow Street, Dundee DD1 5EH, UK; 3 Department of Oncological Sciences, Huntsman Cancer Institute, University of Utah School of Medicine, Salt Lake City, Utah 84112, USA; 4 Howard Hughes Medical Institute, University of Utah School of Medicine, Salt Lake City, Utah 84112, USA; 5 Department of Biology, University of York, York YO10 5DD, UK

## Abstract

Short interspersed nuclear elements (SINEs), such as Alu, spread by retrotransposition, which requires their transcripts to be copied into DNA and then inserted into new chromosomal sites. This can lead to genetic damage through insertional mutagenesis and chromosomal rearrangements between non-allelic SINEs at distinct loci. SINE DNA is heavily methylated and this was thought to suppress its accessibility and transcription, thereby protecting against retrotransposition. Here we provide several lines of evidence that methylated SINE DNA is occupied by RNA polymerase III, including the use of high-throughput bisulphite sequencing of ChIP DNA. We find that loss of DNA methylation has little effect on accessibility of SINEs to transcription machinery or their expression *in vivo*. In contrast, a histone methyltransferase inhibitor selectively promotes SINE expression and occupancy by RNA polymerase III. The data suggest that methylation of histones rather than DNA plays a dominant role in suppressing SINE transcription.

## Abstract (web summary)

Transcription of short interspersed nuclear elements (SINEs) allows retrotransposition that contributes to chromosomal instability. Here, the authors show that methylation of histones, rather than DNA, plays a dominant role in suppressing SINE expression.

## Graphical abstract

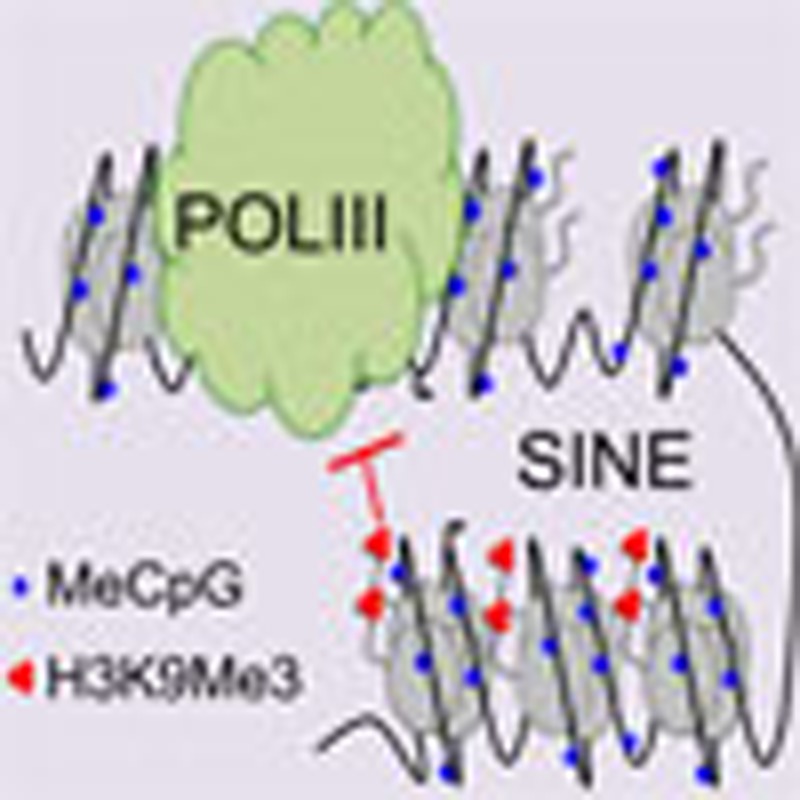

A striking feature of mammalian chromosomes is the large numbers of short interspersed nuclear elements (SINEs) interspersed among the genes. For example, the human genome carries ~10^6^ copies of Alu SINEs that together account for ~11% of total chromosomal DNA. Such SINEs have had a major impact on genomic evolution and place a burden on chromosomal stability through deletions and translocations arising by recombination between non-allelic loci. Indeed, SINEs have caused many instances of human genetic disease^1^,^2^, including α-thalassaemia^3^, hypercholesterolaemia^4^ and neurofibromatosis^5^.

SINEs spread by retrotransposition, which requires their transcripts to be copied into DNA and then inserted into new chromosomal sites. SINE promoters direct transcription by RNA polymerase (pol) III, but expression is extremely weak. For example, the million Alu templates together produce only ~10^3^ transcripts in HeLa cells, whereas the three *7SL* genes per genome produce ~10^6^ transcripts using the same pol III transcription factors as Alu^6^. It was estimated that ~99% of potentially active Alu SINEs with intact promoters may be silenced^7^. Such transcriptional repression is believed to involve packaging the SINEs into chromatin structures that deny access of transcription factors. This may be of great importance, because SINEs can exert boundary effects and regulate messenger RNA (mRNA) synthesis^8^. SINE transcript overexpression can be cytotoxic and cause an untreatable form of human blindness^9^.

SINE transcription is thought to be silenced through methylation of CpG, an important mechanism of gene repression in mammals^10^,^11^. About 7 million of the total 30 million CpG sites in the human genome lie within Alu sequences^12^, with CpG densities ninefold above the average for the human genome in some subfamilies^13^. Heavy methylation is found at the majority of Alu and mouse SINEs^14^,^15^,^16^. That this contributes to transcriptional repression was suggested by an increase in Alu expression after treatment of HeLa cells with the DNA methylation inhibitor 5-azacytidine^6^. Furthermore, methylation of Alu DNA was found to repress its transcription in transient transfections and *in vitro* under some conditions^13^,^15^,^17^. Repression *in vitro* was relieved by miscellaneous methylated competitor DNA, suggesting that it is mediated by one or more *trans*-acting factors with sequence independent affinity for methylated DNA^13^. Examples of such methyl-CpG-binding proteins (MBPs) include MeCP2, MBD1 and MBD2, which operate primarily by directing assembly of repressive chromatin structures that are inaccessible to the transcription machinery^18^. Indeed, MeCP2 was found at Alu SINEs in human cells^19^.

Here we show that human and mouse SINE families are occupied by MeCP2, MBD1 and MBD2. However, SINE transcription is not enhanced by DNA demethylation and release of these MBPs. In contrast, pol III loading and expression of SINEs increases significantly when cells are treated with chaetocin, a selective inhibitor of SUV39 methyltransferases that methylates histone H3 on lysine 9 (H3K9). Endogenous SUV39 associates with SINEs and SINE induction by chaetocin correlates with loss of trimethylated H3K9. The data suggest that methylation of H3K9, rather than DNA, is primarily responsible for suppressing pol III-mediated transcription of genomic SINEs in the cells we have studied.

## Results

### MBPs target SINEs

**Figure 1:**
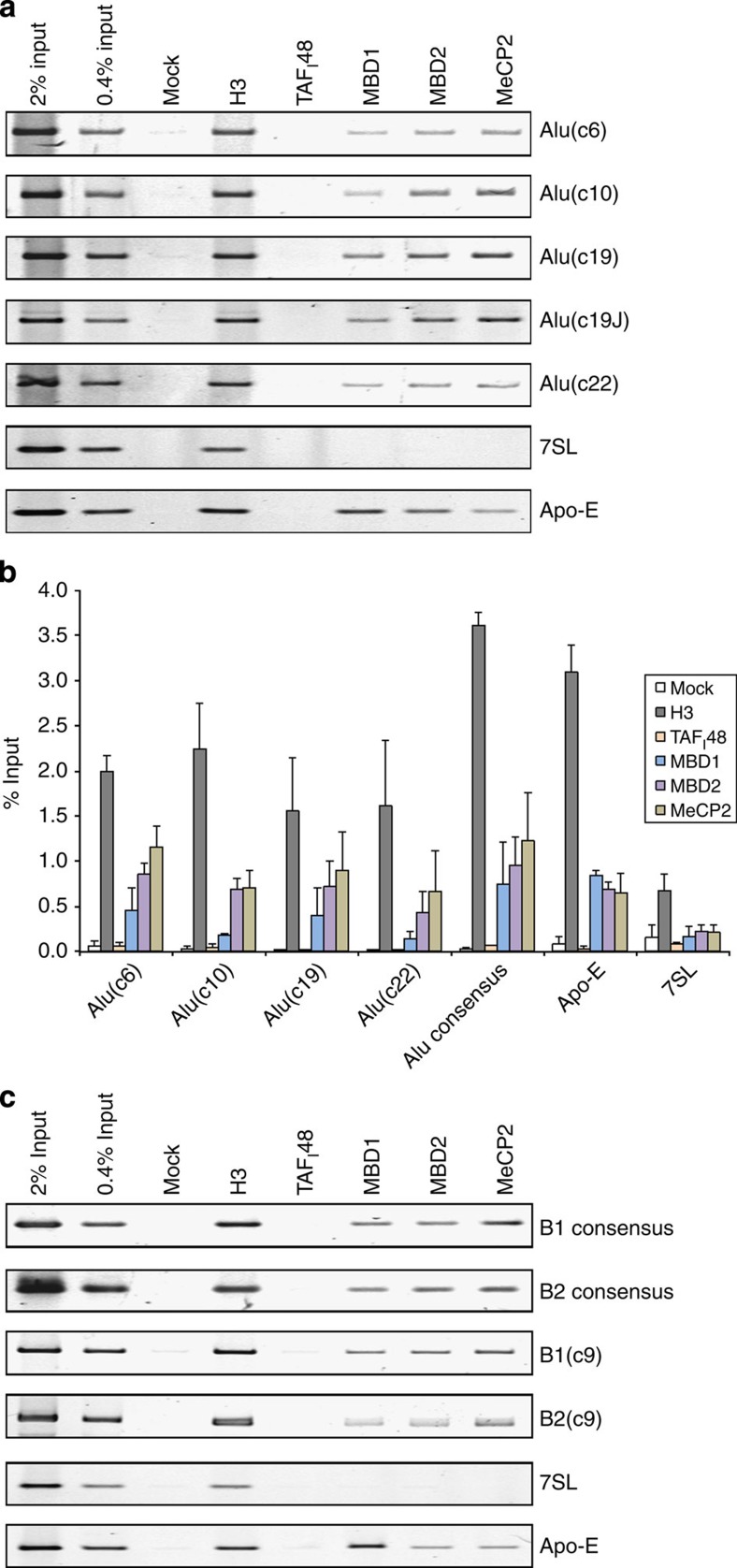
SINEs provide binding sites for MBPs. (**a**) Semiquantitative ChIP assay in HeLa cells showing the binding of MBD1, MBD2 and MeCP2 at Alu loci from chromosomes 6, 10, 19 and 22, as well as 7SL and Apo-E loci. Alu19J is also from chromosome 19. ChIPs for histone H3 and TAF_I_48 provide positive and negative controls, respectively. No antibody was used for the mock sample. (**b**) Mean±s.e.m. of the percentage input bound in two independent ChIP–quantitative PCR assays with HeLa cells and antibodies against histone H3, TAF_I_48, MBD1, MBD2 and MeCP2, as indicated. No antibody was used for the mock samples. Amplifications were carried out using primers for the *Apo-E* and *7SL* genes, Alu PV subfamily consensus sequence and individual Alus on chromosomes 6, 10, 19 and 22, as indicated. (**c**) Semiquantitative ChIP assay in A31 fibroblasts showing binding of MBD1, MBD2 and MeCP2 at B1 and B2 loci, as well as *7SL* and *Apo-E* genes. B1 and B2 consensus primers match ~10^2^ members of the B1 and B2 families, whereas B1(c9) and B2(c9) primers detect unique loci on chromosome 9. ChIPs for histone H3 and TAF_I_48 provide positive and negative controls, respectively.

Consistent with the model that SINEs are subject to MBP-mediated silencing, semiquantitative and quantitative chromatin immunoprecipitation (ChIP)–PCR assays detected MeCP2, MBD1 and MBD2 in HeLa cells at multiple Alu sites, chosen randomly from several chromosomes (Fig. 1a,b). These Alus have 7–13 CpGs each and include an example from chromosome 6 (c6) that is embedded in a cluster of SINEs, ~5 kb from the nearest annotated pol II-transcribed gene. Binding was also detected using consensus primers that recognize ~10^3^ members of an Alu subfamily. Signal intensity is comparable to that obtained with the pol II-dependent *Apo-E* gene, which is known to be silenced by MBPs^20^. Furthermore, binding is selective, as it was not observed at *7SL* genes, the ancestral progenitors of Alu^21^. In contrast to SINEs, 7SL sequences are predominantly unmethylated in genomic DNA.

In rodent genomes, the most abundant SINE families are B1 and B2, instead of Alu^22^. Whereas B1 resembles Alu, both having evolved from 7SL RNA, B2 is unrelated and derived from a transfer RNA (tRNA)^21^,^23^. Nevertheless, both B1 and B2 SINEs are occupied by MeCP2, MBD1 and MBD2 in mouse fibroblasts (Fig. 1c). In each case, clear binding was detected using primers that recognise familial consensus sequences, as well as with unique loci from chromosome 9 (c9). We conclude that abundant mouse and human SINEs are targeted by proteins that mediate transcriptional silencing directed by DNA methylation.

### MBPs do not exclude pol III from SINEs

**Figure 2:**
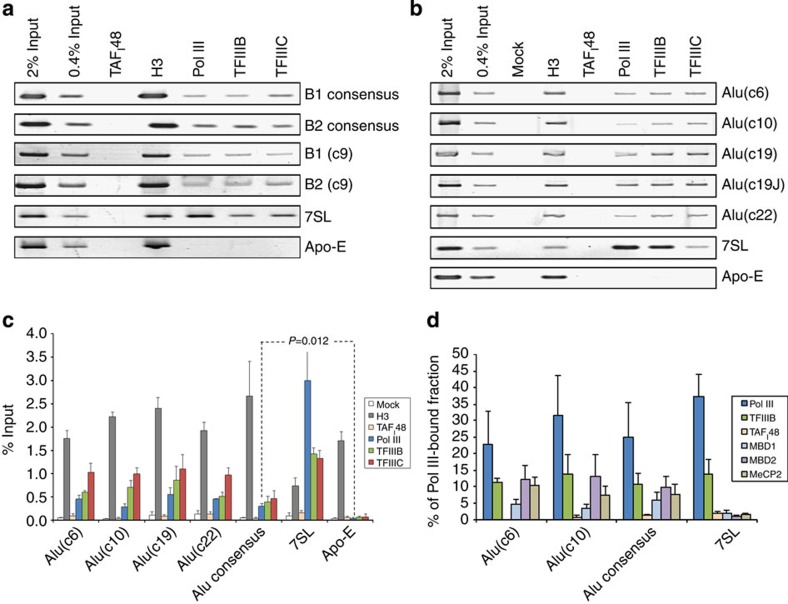
Pol III co-occupies methylated SINEs with MBPs. (**a**) Semiquantitative ChIP assay in A31 fibroblasts showing specific binding of TFIIIB, TFIIIC and pol III to B1 and B2 loci, as well as *7SL*, but not the *Apo-E* gene. Histone H3 and TAF_I_48 provide positive and negative controls, respectively. (**b**) Semiquantitative ChIP assay in HeLa cells showing occupancy of pol III, TFIIIB and TFIIIC at Alu loci from chromosomes 6, 10, 19 and 22, as well as *7SL* and *Apo-E* genes. ChIPs for histone H3 and TAF_I_48 provide positive and negative controls, respectively. No antibody was used for the mock sample. (**c**) Mean±s.e.m. of the percentage input bound in three independent ChIP–quantitative PCR (qPCR) assays in HeLa cells, of the indicated proteins at individual Alu loci from chromosomes 6, 10, 19 and 22, as well as 7SL and Apo-E loci and Alu PV subfamily consensus. ChIPs for histone H3 and TAF_I_48 provide positive and negative controls, respectively. No antibody was used for the mock samples. *P* values are calculated by *t*-test. (**d**) Mean±s.e.m. of four independent sequential ChIP–qPCR assays in which DNA immunoprecipitated from HeLa cells using pol III antibody was reprecipitated using antibodies against pol III, TFIIIB, TAF_I_48 (negative control), MBD1, MBD2 and MeCP2, as indicated. No TAF_I_48 signal was detected on Alu(c6).

Despite the presence of MBPs, pol III was also detected at these SINEs, as were the pol III-specific transcription factors TFIIIB and TFIIIC (Fig. 2a,b). Specificity was confirmed by the lack of pol III, TFIIIB and TFIIIC at the pol II-dependent *Apo-E* gene. ChIP–quantitative PCR demonstrated that pol III detection at consensus Alu elements is approximately sixfold weaker than at the highly active *7SL* genes, but is nevertheless significantly above the background observed on the *Apo-E* gene or with a negative control antibody against TAF_I_48, a pol I-specific transcription factor (Fig. 2c). We conclude that chromosomal SINEs are more accessible to pol III and its associated transcription factors than had previously been thought.

The clear detection of both pol III and MBPs at these loci could be explained if a mixed cell population was present, with the same SINE occupied by MBPs in some cells and by pol III machinery in others. Sequential ChIP assays were used to test this hypothesis. DNA occupied by pol III was immunoprecipitated and the samples were then re-immunoprecipitated with antibodies to MeCP2, MBD1 and MBD2, as well as TFIIIB and pol III again as positive controls. For each MBP tested, Alu DNA was recovered at the levels well above background, indicating that all three MBPs can interact with a SINE at the same time as pol III (Fig. 2d). This was also the case for B1 and B2 SINEs and when TFIIIC was chromatin immunoprecipitated first instead of pol III (Supplementary Fig. 1).

Co-occupancy of MBPs with pol III and its associated factors suggests that the latter can bind to methylated DNA. This possibility is supported by ChIP–chop assays, in which DNA recovered from ChIP samples is subjected to restriction digestion at sequences of potential methylation^24^. As expected, Alu DNA associated with MBD2 or MeCP2 was digested more readily by the methylation-insensitive restriction enzyme MspI than by its methylation-sensitive isoschizomer HpaII (Supplementary Fig. 2). The same was found for Alu DNA bound by pol III or TFIIIC, demonstrating that these proteins also interact *in vivo* with methylated SINE DNA.

**Figure 3:**
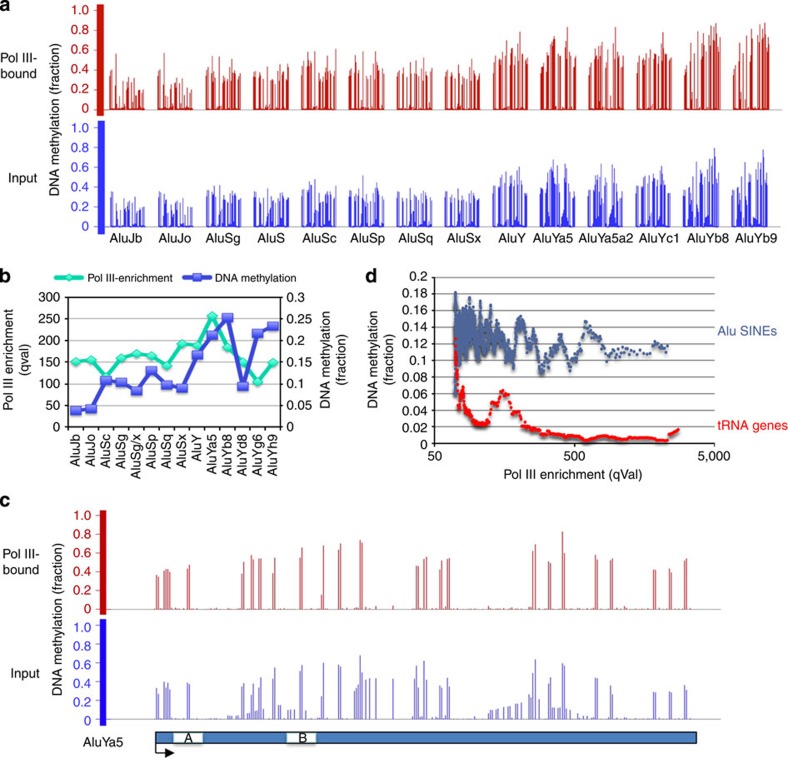
Genome-wide methylation analysis of pol III-bound Alu DNA. (**a**) Comparison of the fraction of methylation of the indicated Alu subfamilies between genomic DNA (input) and pol III-bound DNA, as determined by ChIP-BS-Seq. The height of a vertical line indicates the fraction methylated at a particular C within the subfamily consensus sequence. (**b**) Genome-wide comparison of pol III occupancy and DNA methylation of Alu subfamilies. (**c**) Comparison of the extent of methylation of the AluYa5 subfamily between genomic DNA (input) and pol III-bound DNA. The height of a vertical line indicates the fraction methylated at a particular C within the subfamily consensus sequence. A- and B-block promoter elements are indicated. (**d**) Pol III occupancy plotted against DNA methylation for Alu SINEs (blue) and tRNA genes (red). Data are plotted as a moving average with 25 data points per window.

Bisulphite sequencing of a pol III-bound Alu following ChIP demonstrated the high levels of CpG methylation (Supplementary Fig. 3). To examine this globally, we applied the recently reported genome-scale ChIP-bisulphite-sequencing (ChIP-BS-Seq) technique to assess DNA methylation status of pol III-bound DNA^25^. Using this method, we found that the methylation levels are comparable for input DNA and for Alu DNA cross-linked to pol III, showing that DNA methylation does not deter occupancy (Fig. 3a). This is the case for all Alu classes, including the relatively young AluY subclasses that are most active in retrotransposition. Indeed, the AluY SINEs generally show significantly higher DNA methylation than the older AluS and AluJ classes, and also higher pol III occupancy, although the latter does not reach statistical significance (Supplementary Data 1 provides the full data set). The AluYa5 subclass has the highest occupancy by pol III, despite being one of the most strongly methylated (Fig. 3b). Inverse correlation was not found between pol III occupancy and DNA methylation. Focusing on the B-block region, which provides the primary, high-affinity binding site for TFIIIC, comparison of the three main classes revealed CpG methylation of 55–65% for AluY, compared with 30–50% for AluS and 13% for AluJ, the oldest Alu class. It is striking that the A- and B-block promoter regions that direct transcription complex assembly contain CpG dinucleotides that are methylated in pol III-bound DNA at comparable levels to the input DNA (Fig. 3c; Supplementary Fig 4). Indeed, 12.9% of Alu SINEs show statistically significant enrichment for CpG methylation in the pol III ChIP relative to the input DNA, whereas this is only the case for 0.4% of tRNA genes. Overall, Alu SINEs have much higher levels of CpG methylation than tRNA genes, irrespective of whether they are occupied by pol III (Fig. 3d). Collectively, the data indicate that template methylation does not prevent access of pol III to SINEs. Furthermore, pol III can be detected throughout the length of occupied SINEs (Supplementary Fig 5).

**Figure 4:**
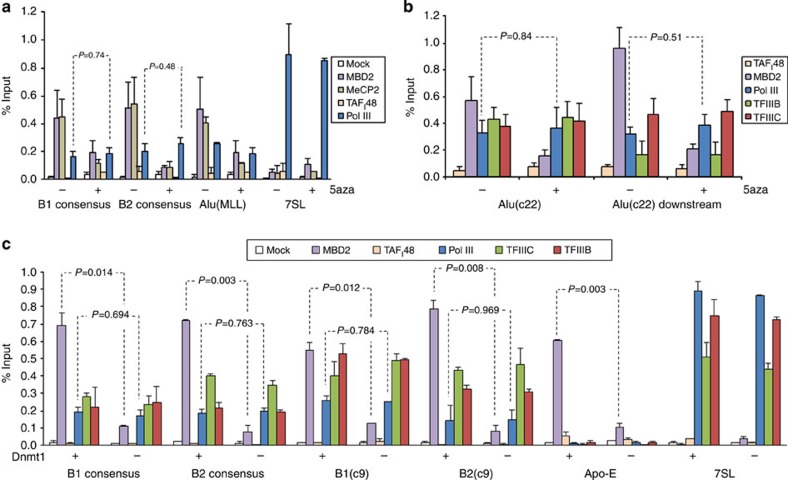
DNA methylation does not prevent pol III occupancy of SINEs. (**a**) Percentage input bound in three independent ChIP–quantitative PCR (qPCR) assays with mouse ES cells treated for 16 h with (+) or without (−) 5-azacytidine, showing occupancy of MBD2, MeCP2 and pol III at 7SL, B1 and B2 loci, as well as an Alu inserted onto chromosomes 14 and 17. ChIPs for TAF_I_48 and without antibody (mock) provide negative controls. (**b**) Percentage input bound in three independent ChIP–qPCR assays with HeLa cells treated for 72 h with (+) or without (−) 5-azacytidine, showing the binding of MBD2, TFIIIB, TFIIIC and pol III to DNA centred over the body of Alu(c22) or 200 bp downstream. The resolution of this assay is limited by the size of the genomic DNA fragments (~500 bp). (**c**) Percentage input bound in two independent ChIP–qPCR assays with matched Dnmt1^+/+^ and Dnmt1^−/−^ fibroblasts showing occupancy of MBD2, TFIIIB, TFIIIC and pol III at B1 and B2 loci, as well as *7SL* and *Apo-E* genes. ChIPs for TAF_I_48 and without antibody (mock) provide negative controls. Error bars indicate s.e.m. and all *P* values are calculated by *t*-test.

We tested the effect on pol III occupancy of treating cells with 5-azacytidine at concentrations that elicit global genomic demethylation. The efficacy of the treatment was confirmed by a clear decrease in MBP binding *in vivo* (Fig. 4a,b) and was confirmed *in vitro* following extraction of genomic DNA (Supplementary Fig. 6). However, pol III detection at Alu, B1 and B2 SINEs was not increased, providing further evidence that DNA methylation does not preclude access of pol III to SINEs (Fig. 4a,b). Detection of TFIIIB and TFIIIC at SINEs is also unaffected by 5-azacytidine (Fig. 4b). To address the possibility that CpG demethylation might affect the distribution of pol III along its template, we included primers located downstream of an Alu. As expected, the signal for TFIIIB is weaker using the downstream primers, whereas robust signals from pol III and TFIIIC are maintained; this is consistent with the established localization of these proteins^26^. As with other primer sets, the pol III signal with the downstream primer set was not significantly affected by 5-azacytidine (Fig. 4b). Although the shortness of SINEs limits the study of polymerase distribution using this technology, the available data do not support the possibility that demethylation alters pol III positioning along SINE DNA.

We also found comparable levels of SINE occupancy by pol III between fibroblasts with or without targeted disruption of the gene encoding Dnmt1 (Fig. 4c), despite the fact that the Dnmt1-knockout cells have <5% of the normal level of DNA methylation^27^. The ChIP signals from TFIIIB and TFIIIC on SINEs are also comparable in the presence or absence of Dnmt1. Although expression of MBD2 and MeCP2 is undiminished in the knockout cells, their binding to B1 and B2 loci is significantly compromised (Fig. 4c; Supplementary Fig. 7). This observation excludes the unlikely possibility that methylation of B1 or B2 DNA is immune to loss of Dnmt1. We conclude that pol III and its associated transcription factors are not prevented from accessing SINEs *in vivo* by DNA methylation or the presence of various MBPs.

### SINE repression does not rely on DNA methylation

**Figure 5:**
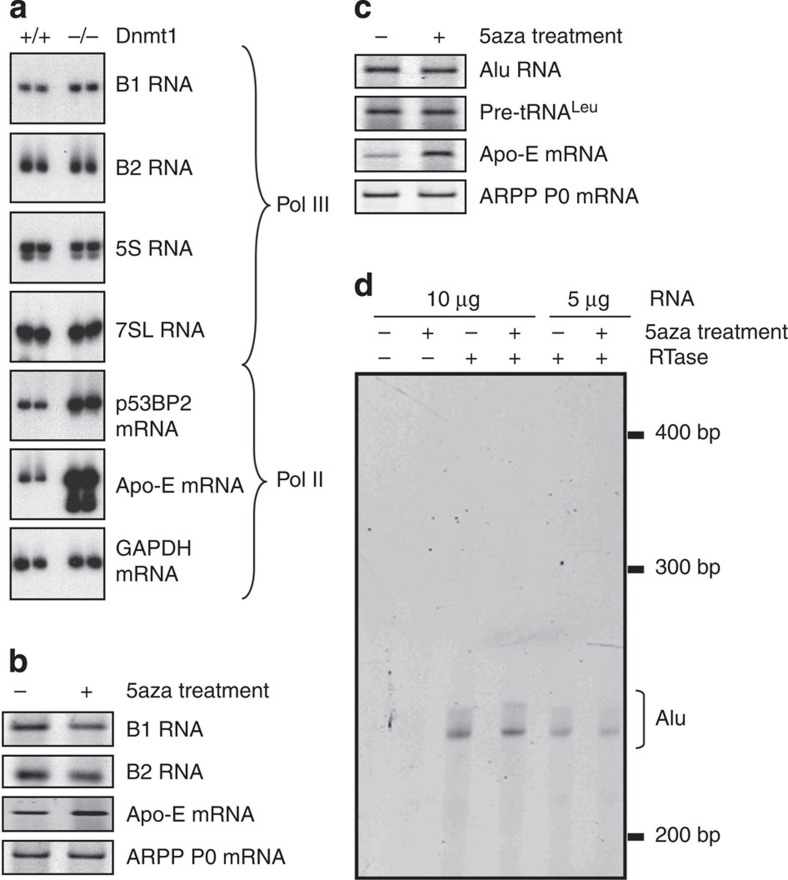
SINE expression is not stimulated by loss of DNA methylation. (**a**) Analysis by semiquantitative RT–PCR of expression levels of the indicated transcripts in matched Dnmt1^+/+^ and Dnmt1^−/−^ fibroblasts. Duplicate samples are shown for both cell types. Apo-E and p53BP2 mRNAs provide controls that have been documented as being suppressed by DNA methylation. GAPDH mRNA provides a loading control. (**b**) Analysis by semiquantitative RT–PCR of expression levels of the indicated transcripts in mouse ES cells treated for 16 h with (+) or without (−) 5-azacytidine. Apo-E mRNA provides a control that has been documented as being inhibited by DNA methylation. ARPP P0 mRNA provides a loading control. (**c**) Analysis by semiquantitative RT–PCR of expression levels of the indicated transcripts in HeLa cells treated for 72 h with 5-azacytidine. Apo-E mRNA provides a control that has been documented as being inhibited by DNA methylation. ARPP P0 mRNA provides a loading control. (**d**) Analysis by primer extension of Alu transcripts in the RNA from Fig. 5c. Bracket indicates ~240 bp products that initiate at the principle pol III start site of Alu. Reverse transcriptase was omitted from the reactions in lanes 1 and 2. To confirm that the assay was not saturated, raising the amount of template RNA from 5 (lanes 5 and 6) to 10 μg (lanes 3 and 4) is shown to give a stronger signal. Alu, B1 and B2 RT–PCRs were performed with Alu, B1 and B2 consensus primers, respectively (Supplementary Table 1).

Dnmt1-knockout fibroblasts show little or no increase in B1 or B2 RNA (Fig. 5a), despite robust induction of the *Apo-E* and *p53BP2* genes, both of which are subject to methylation-dependent silencing^20^,^28^. This is consistent with a previous report that the B1 transcript levels are unchanged relative to wild type in Dnmt1^−/−^ or Dnmt3ab^−/−^ embryonic stem (ES) cells, in which CpG methylation of B1 DNA is reduced by approximately two- to fourfold^29^.

Because SINEs are often located within introns and untranslated regions of protein-coding genes, SINE sequences are found within many longer pol II-derived RNAs. As this can complicate interpretation, we applied α-amanitin at a concentration that inhibits pol II while allowing transcription by pol III. Again we found no evidence that B1 or B2 expression is elevated in Dnmt1^−/−^ fibroblasts relative to wild type (Supplementary Fig. 8). This is consistent with the unchanged occupancy by pol III, TFIIIB and TFIIIC, as determined by ChIP (Fig. 4c).

As knockout cells have the potential to adapt to permanent loss of a component, we also used 5-azacytidine to promote DNA demethylation in wild-type cells. This caused no increase in the expression of B1 or B2 transcripts in ES cells (Fig. 5b). Similarly, Alu expression was not increased when HeLa cells were treated with 5-azacytidine (Fig. 5c). In both cases, efficacy of the treatment was confirmed by induction of Apo-E mRNA in the same samples. It was also demonstrated by the release of MBD2 and MeCP2 from B1, B2 and Alu SINEs (Fig. 4a,b). The fact that pol III occupancy did not increase is consistent with the absence of increased expression. Indeed, small but consistent decreases were seen in pol III binding and transcript expression; the reason for this has not been pursued.

PCR with reverse transcription (RT–PCR) does not distinguish between transcripts synthesized by pol III from a SINE’s own promoter and pol II-derived RNAs that contain SINE sequences due to the fortuitous presence of these transposable elements in protein-coding genes. We were concerned that a change in pol III-mediated transcription of SINEs might be masked in this assay by constant expression of SINE-containing pol II transcripts. However, the use of a pol III-specific inhibitor established that this is not the case and that the contribution of pol III to the total levels of SINE RNA is detectable using our RT–PCR assay (Supplementary Fig. 9). Nevertheless, primer extension was used to focus on transcripts that initiate specifically at the pol III transcription start site and distinguish these from read-through transcripts initiated upstream^6^. Transcripts detected by this approach are Alu-specific and resistant to doses of α-amanitin that inhibit pol II transcription (Supplementary Figs 10,11). Primer extension assays showed that 5-azacytidine treatment makes no significant difference (*P*=0.54, *t*-test) to the expression of Alu RNA that initiates at the pol III start site, although significantly (*P*=0.000065, *t*-test) enhancing Apo-E expression (Fig. 5d; Supplementary Fig. 12). We conclude that DNA methylation does not suppress the occupancy or activity of pol III at SINEs, despite attracting MBPs to these loci.

### SUV39H1 suppresses pol III occupancy and expression of SINEs

**Figure 6:**
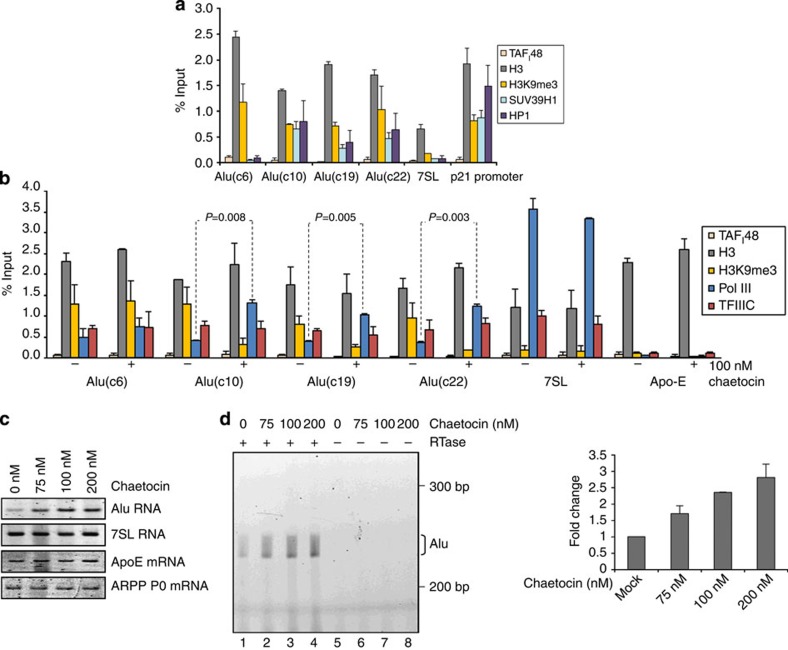
SUV39H1 inhibits pol III loading and expression of some SINEs. (**a**) Mean±s.e.m. of the percentage input bound in two independent ChIP–quantitative PCR (qPCR) assays with HeLa cells, showing occupancy of H3, TAF_I_48, H3K9me3, SUV39H1 and HP1 at Alu loci, *7SL* and *p21* (positive control) genes. (**b**) Mean±s.e.m. of the percentage input bound in two independent ChIP–qPCR assays with HeLa cells treated for 24 h with vehicle (−) or 100 nM chaetocin (+), showing occupancy of H3, TAF_I_48, H3K9me3, pol III and TFIIIC at Alu loci, *7SL* and *Apo-E* genes. (**c**) Semiquantitative RT–PCR analysis of expression levels of indicated transcripts in HeLa cells treated for 24 h with indicated concentrations of chaetocin. (**d**) Analysis by primer extension of expression levels of Alu transcripts initiated from pol III start site in HeLa cells treated for 24 h with indicated concentrations of chaetocin. Reverse transcriptase was omitted from reactions in lanes 5–8. Right panel shows mean±s.d. of fold change in Alu expression in two independent experiments quantified using ImageJ. All *P* values are calculated by *t*-test.

Multiple Alu sequences were recovered in a low-throughput genomic screen of sites bound by K9-methylated histone H3 (ref. 30). Indeed, 68% of the clones isolated either contained Alu sequence or were <200 bp from one^30^. This constitutes a strong enrichment, since Alu provides ~11% of genomic DNA. Consistent with this, the Alu loci we tested show clear trimethylation of histone H3 lysine 9 (H3K9me3; Fig. 6a; Supplementary Fig. 13). In contrast, H3K9me3 is close to background at active *7SL* genes.

One of the enzymes that methylates H3K9 is SUV39H1 (ref. 31). That this histone methyltransferase might mediate SINE repression was suggested by the fact that a dominant-negative Suv39h mutant stimulates the expression of B1 and B2 RNA^29^. We detected SUV39H1 at several Alu loci (Alus c10, c19 and c22), but an example was also found (Alu(c6)) where H3K9me3 does not correlate with detection of SUV39H1 (Fig. 6a; Supplementary Fig. 13). SUV39H1 was also detected at the p21 promoter, one of its documented targets^32^, but was close to background at *7SL* genes. The combined presence of methylated H3K9 and SUV39H1 has been shown to recruit HP1 (ref. 33). Accordingly, HP1 was detected at the p21 promoter and Alu loci with SUV39H1 and H3K9me3, but not at 7SL or Alu(c6) where SUV39H1 is absent. When cells were treated with chaetocin, a selective inhibitor of the SUV39 family^34^, H3K9me3 levels decreased specifically at the Alu sites where SUV39H1 was detected (Fig. 6b; Supplementary Fig. 14). We conclude that SUV39H1 trimethylates H3K9 at some Alu loci, but is not unique in this regard. SUV39H1 is one of several H3K9 methyltransferases^35^,^36^.

At tRNA genes, H3K9me3 shows a strong inverse correlation with pol III occupancy^37^,^38^. Consistent with this, pol III binding to Alu DNA is increased by chaetocin, but only at the loci where H3K9me3 decreases (Fig. 6b; Supplementary Fig. 14). The action of chaetocin is specific, as it increases pol III binding significantly without affecting the binding of TFIIIC or total histone H3. Furthermore, these effects were only observed at loci where SUV39H1 was detected. Elevated Alu expression was observed in chaetocin-treated cells (Fig. 6c) and primer extension demonstrated increased use of the Alu pol III initiation sites (Fig. 6d). The fact that Alu RNA increases when 7SL RNA does not provides evidence that detection of Alu expression is not masked in our assays by the highly abundant 7SL transcript.

**Figure 7:**
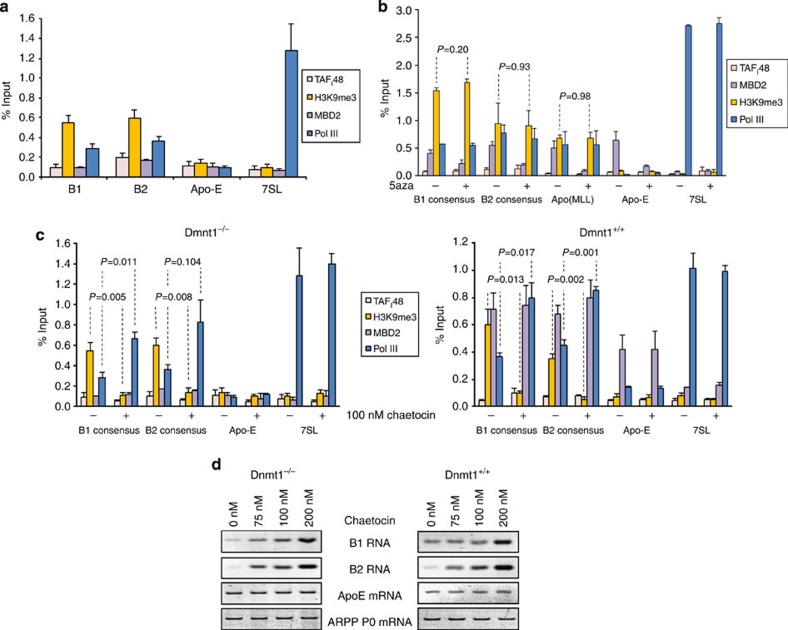
SINE repression by H3K9Me3 does not require DNA methylation. (**a**) Percentage input bound in three independent ChIP–quantitative PCR (qPCR) assays with Dnmt1^−/−^ fibroblasts assessing binding of TAF_I_48, H3K9me3, MBD2 and pol III at 7SL, B1, B2 and Apo-E loci. H3K9me3 data were normalized against total H3. For clarity, Fig. 7a displays a subset of the data shown in Fig. 7c. (**b**) Percentage input bound in two independent ChIP–qPCR assays of ES cells treated for 16 h with (+) or without (−) 5-azacytidine, assessing binding of TAF_I_48, MBD2, H3K9me3 and pol III at 7SL, B1 and B2 loci, as well as an Alu inserted onto chromosomes 14 and 17. H3K9me3 data were normalized against total H3. (**c**) Percentage input bound in three independent ChIP–qPCR assays of matched Dnmt1^−/−^ and Dnmt1^+/+^ fibroblasts treated for 24 h with (+) or without (−) 100 nM chaetocin, assessing binding of TAF_I_48, H3K9me3, MBD2 and pol III at 7SL, B1 and B2 and Apo-E loci. H3K9me3 data were normalized against total H3. (**d**) Semiquantitative RT–PCR analysis of expression levels of indicated transcripts in matched Dnmt1^+/+^ and Dnmt1^−/−^ fibroblasts treated for 24 h with indicated concentrations of chaetocin. Error bars indicate s.e.m. and all *P* values are calculated by *t*-test.

The data suggest that H3K9me3 is inhibitory to pol III recruitment and therefore contributes to suppression of SINE transcription. To test whether this mark on SINEs depends on methylation of their DNA, we examined its presence in fibroblasts with targeted disruption of Dnmt1. Despite the >95% decrease in DNA methylation^27^, H3K9me3 was clearly detected at B1 and B2 SINEs in the Dnmt1-null cells (Fig. 7a). Furthermore, 5-azacytidine treatment did not significantly decrease the levels of H3K9me3 or SUV39H1 on B1, B2 or Alu SINEs in ES or HeLa cells, although MBD2 binding decreased (Fig. 7b; Supplementary Figs 15,16). In contrast to 5-azacytidine, chaetocin treatment stimulated pol III occupancy and the expression of both B1 and B2 in the presence or absence of Dnmt1 (Fig. 7c,d). These data suggest that SUV39H1 inhibits recruitment of pol III and transcription of SINEs independently of DNA methylation.

### DNA methylation suppresses translocation between Alu SINEs

**Figure 8:**
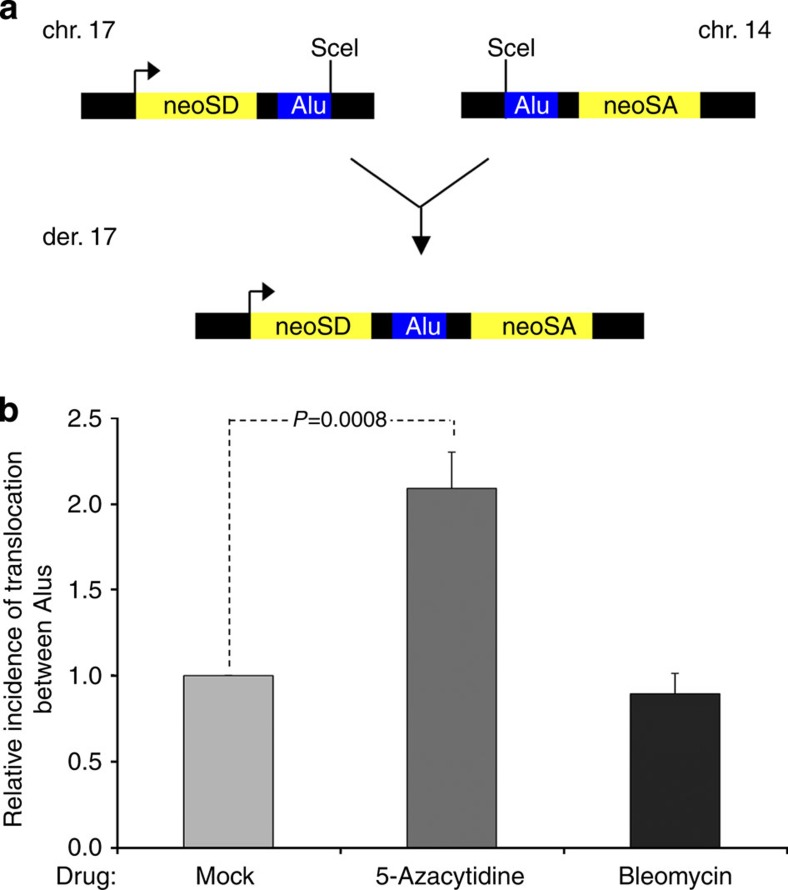
5-azacytidine stimulates translocation between Alus. (**a**) Schematic illustration of system used to induce and detect chromosomal translocations at Alu elements. (**b**) Mean±s.d. from three independent experiments measuring the relative incidence of translocation between Alu elements located on chromosomes 14 and 17 in ES cells treated with 5-azacytidine or bleomycin, relative to cells without drug. *P* values are calculated by *t*-test.

Cytosine methylation has been shown to inhibit homologous recombination between satellite DNA and its deficiency can cause human genetic disease^39^. We therefore considered whether SINE methylation might protect the genome by suppressing DNA rearrangements. This issue is important, because Alu elements are so frequent in the human genome (one every ~3 kb on average), are concentrated in gene-rich regions and are highly homologous to each other. We tested whether demethylation promotes rearrangements of SINEs using an assay that detects translocations between two Alu copies targeted to chromosomes 14 and 17 in mouse ES cells^40^. Although much less common than recombination between proximal Alu pairs, interchromosomal translocations can be life-threatening^1^. For example, the Alu used in this assay comes from intron 1 of the human *MLL* gene and participates in MLL duplications that are found in patients with acute myeloid leukaemia^41^. ChIP confirmed that this ectopic Alu is selectively bound by pol III and MBPs in mouse ES cells, as seen for multiple Alus in human cells (Fig. 4a; Supplementary Fig. 17). Translocation is initiated using I-SceI endonuclease to target a double-strand break specifically to the Alu loci. It is detected using a split neomycin phosphotransferase gene (*neo*) with an MLL Alu positioned downstream of a *neo* splice donor site on chromosome 17 and another upstream of a *neo* splice acceptor site on chromosome 14; reciprocal translocations between the two sites generate functional *neo*^*+*^ genes that can be detected by screening for neomycin-resistance (Fig. 8a). The molecular basis of this resistance was confirmed by detection of translocation-specific PCR products (Supplementary Fig. 18).

Treatment of these cells with 5-azacytidine releases MeCP2 and MBD2 from the MLL-derived Alu elements (Fig. 4a). It also causes a significant increase in translocations (Fig. 8b; Supplementary Data 2). Genomic translocations result from chromosomal breakage and misrepair, especially of double-strand breaks. We therefore tested whether the Alu-mediated translocations are also induced by bleomycin, an agent that induces such breaks efficiently but does not cause demethylation. In contrast to 5-azacytidine, bleomycin failed to stimulate Alu translocation in this system. This suggests that the increased rearrangements induced by 5-azacytidine reflect its demethylating activity.

## Discussion

Retrotransposons provide hotspots for genomic recombination. They contain most of the methylated cytosines in human DNA and this methylation is thought to suppress transposition to promote genomic integrity^42^. Demethylation of satellite DNA has been linked to chromosomal rearrangements and human genetic disease^39^. Because SINEs are concentrated in and around the protein-coding genes, their translocation can be especially disruptive, as illustrated by many oncogenic rearrangements^2^,^43^,^44^. For example, acute myeloid leukaemias have arisen through recombination between intronic Alus, leading to partial duplication of the *MLL* gene^45^,^46^,^47^. Using a model of such lesions, we found that interchromosomal translocation between a pair of Alu SINEs is suppressed by DNA methylation to a significant degree (*P*=0.0008, *t*-test). SINE-mediated rearrangements may, therefore, contribute to the elevated mutation rates found in most cancer cells where genomic hypomethylation is widespread^48^,^49^,^50^. Indeed, hypomethylation of Alu DNA was found to correlate (*P*=0.008) with genomic instability in human lung carcinomas^51^.

Translocations between Alus arise when misrepair of a double-strand break occurs through either of two pathways—non-homologous end-joining or single-strand annealing^40^. The latter predominates in the assay we have used. This remains the case after treatment with 5-azacytidine (Supplementary Fig. 18), suggesting that demethylation affects the frequency of these translocations, but not the repair pathway employed. However, demethylation may also influence this assay indirectly by affecting expression of the DNA repair machinery or the rearranged reporter gene. We therefore regard this as an interesting but preliminary observation. Further mechanistic analysis will be required to establish whether methylation of Alu DNA affects rearrangement frequency directly.

Although it may influence translocation, directly or indirectly, SINE DNA methylation does not prevent transcription factor occupancy or function under the conditions we have studied. This was unexpected and contrasts with conclusions drawn from several previous studies. DNA methylation was reported to inhibit TFIIIC binding *in vitro*, but the DNA fragment tested lacked clear promoter elements^52^. Increased Alu content was found in RNA from lung cancer cell lines after treatment with 5-aza-2′-deoxycytidine, but that study did not distinguish between pol III products and Alu sequences embedded in pol II transcripts^51^. However, the expression of pol III-initiated Alu RNA was shown to be substantially elevated after 8 days treatment of HeLa cells with 5-azacytidine^6^. Indirect effects might have caused this response, given the long duration of drug treatment. The authors confirmed that Alu loci were demethylated^6^, but this does not prove that the Alu demethylation was responsible directly for the observed expression changes. Indeed, a study with U2OS cells found that pol III transcripts are induced after 5 days treatment with 5-aza-2′-deoxycytidine, but not after 2 days; in contrast, the *p16* gene, which served as positive control, was induced >100-fold in the 2-day-treated samples^53^. Although Alu transcripts were not examined, each of five other pol III products tested showed induction after 5 days but not after 2 days^53^. These data provide clear evidence for secondary effects on the pol III machinery after long-term treatment with demethylating agents. We used shorter drug treatments (16–72 h), which were sufficient to cause SINE demethylation and release of MBPs, but did not enhance expression. Another study found that methylation of a chimeric 7SL/Alu reporter inhibited its expression in transfected cells; contrary to expectation, cotransfection of a MeCP2 overexpression vector relieved this repression, rather than compounding it^17^. This surprising result may reflect indirect effects or the fact that transiently transfected reporters do not adopt the chromatin structures of endogenous genes^54^. Clear evidence that methylation of Alu DNA can directly inhibit its transcription by pol III has been obtained using assays *in vitro*^13^,^15^. However, the inhibitory effect *in vitro* was only seen at low template concentrations and was absent when more Alu DNA was used^13^,^15^. Our data suggest that the immunity to repression seen with higher template concentrations mimics the situation *in vivo*. Nuclear concentrations are orders of magnitude higher than those employed for *in vitro* transcription reactions. Perhaps, high local concentrations and the context of supercoiled chromatin allow pol III to cope better with methylated templates *in situ* than in dilute reactions *in vitro*. The failure of DNA methylation to silence pol III transcription in cells is established here through both genetic and pharmacological approaches. Furthermore, unlike the previous studies, our expression data are supported strongly by direct assessment of pol III occupancy on SINEs and of the methylation state of pol III-bound SINE DNA. The possibility remains, nevertheless, of a more dominant role for DNA methylation in different cell types or conditions.

Although DNA methylation appears not to play a major role in excluding pol III from its templates under the conditions we have studied, trimethylation of H3K9 is clearly involved. This modification was already known to be inversely correlated with pol III occupancy and expression of tRNA genes^37^. We found that inhibition of SUV39H1 selectively reduces H3K9 trimethylation and stimulates pol III loading onto a subset of SINEs, while raising expression of SINE transcripts substantially. An exception is Alu(c6), where SUV39H1 inhibition did not significantly alter H3K9me3 or pol III detection (Fig. 6b); this suggests that some SINEs are targeted by H3K9 methyltransferases other than SUV39H1. Local environment might dictate which methyltransferase operates at individual SINEs.

In contrast to pol III, the binding of TFIIIC to SINEs is not enhanced by inhibition of SUV39H1 (Fig. 6b). Indeed, TFIIIC detection at SINEs is comparable to that at *7SL* genes, which attract much higher levels of pol III (Figs 2a,b and 6b). These observations are consistent with the reports that TFIIIC can overcome chromatin-mediated repression *in vitro*^55^,^56^. TFIIIC is responsible for promoter recognition on most pol III-transcribed genes, including SINEs, and then recruits TFIIIB and pol III by protein–protein interactions^26^,^57^. We believe it is the pol III recruitment step specifically that, rather than promoter recognition, is inefficient *in vivo* at Alu, B1 and B2 SINEs. Thus, quantification of multiple ChIP experiments revealed a significantly higher ratio of pol III to TFIIIC on 7SL relative to Alu (*P*=0.007, *t*-test) in human cells and on 7SL relative to B1 (*P*=0.002, *t*-test) and B2 (*P*=0.003, *t*-test) in mouse cells. Since pol III itself has minimal DNA sequence specificity, it is likely to be the chromatin landscape of SINEs that discourages its recruitment. Our data suggest that H3K9me3 is a feature of SINE chromatin that impedes pol III recruitment and transcription.

Despite the inhibitory effects of chromatin, we were able to detect pol III at ~1,400 Alu SINEs across the human genome, although with generally weaker binding than observed at active tRNA genes. This contrasts with several previous studies that recorded pol III occupancy of very few Alu SINEs^38^,^58^,^59^. For example, only 13 Alu dimer loci were reported as pol III bound in ref. 38. The discrepancy may reflect a number of technical differences, including the choice of statistical threshold. Antibody efficiency may be especially important, as the pol III antibody used in our current study gives unusually high enrichment. Using the same pol III antibody as in our current study, Moqtaderi *et al*.^60^ estimated that ~1,000 SINEs were pol III occupied in K562 cells, although the levels of pol III at these loci were again generally much lower than at tRNA genes^60^. There is only ~2% overlap between the sets of Alu loci occupied by pol III in the two studies, which used different cell types. This suggests that access to SINEs may not only be limited, but also highly variable according to conditions. As well as transcriptional suppression, degradation of SINE RNA provides additional protection against accumulation of their transcripts^9^.

## Methods

### Cell culture

HeLa cells and A31 fibroblasts were kindly provided by Peter Rigby. Maria Jasin generously provided Hom Alu mouse ES cells, in which an Alu element from intron 1 of the human *MLL* gene is carried within the *Pim1* locus of chromosome 17 and the *Rb* locus of chromosome 14 (ref. 40). Cells were cultured in DMEM supplemented with 10% FCS, 2 mM L-glutamine, 100 U ml^−1^ penicillin and 100 U ml^−1^ streptomycin. For the Dnmt1^+/+^ and Dnmt1^−/−^ fibroblasts, kindly provided by Adrian Bird, Howard Cedar and Ittai Ben-Porath, medium was supplemented with 2 mM sodium pyruvate, 1% non-essential amino acids and 0.01% β-mercaptoethanol; these cells have a p53-null background^27^. 5-azacytidine (Sigma) was used at 4 μM for 16–72 h, as indicated. Bleomycin (Calbiochem) was used at 5 μg ml^−1^ for 16 h. Treatment with chaetocin (Sigma) was for 24 h and with α-amanitin (Sigma) was as indicated.

### ChIP assays

ChIP–PCR and sequential ChIP–PCR assays were performed as previously described^61^. Protein–DNA complexes were cross-linked using 1% formaldehyde for 10 min at room temperature and the reaction was quenched with 0.125 M glycine. Cells were washed with PBS 0.5% NP-40 and lysed by incubation in 1 M NaCl PBS/NP-40, followed by 0.1 M NaCl and 10 mM TE/NP-40. The nuclei were then cleaned by centrifugation through a 100 mM sucrose cushion. The chromatin was sheared by water bath sonication using a Bioruptor (Diagenode) to obtain a median fragment size of 500 bp. Input samples were removed and immunoprecipitation was performed overnight in 150 mM NaCl TE/NP-40 using 5 μg antibody bound to protein A/G sepharose. Immunoprecipitates were washed twice in RIPA buffer, twice in 250 mM LiCl 0.5% Na-deoxycholate TE/NP-40, twice in TE and elution was performed in TE/1%SDS. The eluate from the first ChIP was diluted 1:10 before a sequential ChIP. Proteinase K digestion was performed overnight at 42 °C. DNA was purified with QIAquick PCR purification kit (Qiagen), according to the manufacturers’ instructions.

Serial dilutions of the input chromatin were used to confirm that PCRs were within a linear range. *P* values were obtained using a two-tailed unpaired *t*-test. PCR primers and amplification conditions are described in Supplementary Table 1. Individual loci were specified using primers for unique sequence flanking a particular SINE. The B1(c9) and B2(c9) primers detect unique sequences on c9 within a cluster that has no recognized pol II transcription unit within 5 kb. B1 and B2 primers detect subgroups of ~10^2^ family members each. The Alu consensus primers match the PV subfamily that has ~10^3^ members.

For ChIP–chop assay^24^, ChIP DNA was spiked with 100 ng of unmethylated PCR product containing a HpaII/MspI site (to normalize for digestion efficiency) and then digested for 1 h with HpaII or MspI.

Antibodies used were M9317 against MeCP2 and M7318 against MBD2 (Sigma), IMG-306A against MBD1 (Imgenex), ab1791 against histone H3 (Abcam), 07-108 against histone H4 and 05-615 against SUV39H1 (Upstate), 9754S against H3K9me3 (Cell Signaling), sc-25366 against SUV39H1, sc-28735 against HP1, sc-25365 against TFIIA and sc-6571 against TAF_I_48 (Santa Cruz Biotechnology), 1900 against the RPC155 subunit of Pol III^62^, 128 against the Brf1 subunit of TFIIIB, 4286 against the 110 kD subunit of TFIIIC^63^ and Ab7 against the 220 kD subunit of TFIIIC^37^. Uncropped scans are shown in Supplementary Fig. 19.

### ChIP-BS-Seq library preparation and sequencing

For HeLa input DNA, two libraries were prepared from the same input DNA: one with starting quantity of 10 ng, following the Illumina ChIP-Seq library protocol, with the addition of bisulfite conversion after addition of adaptors but before amplification; and one following the Illumina Bisulfite Sequencing protocol, including the recommended starting amount. All libraries included addition of 2% sheared lambda DNA to control for bisulfite conversion. The Qiagen EpiTect kit was used for bisulfite conversion, with four periods of incubation at 60 °C (for 25, 85, 175 and 120 min), each preceded by denaturation for 10 min at 98 °C. Size selection was not performed for the amplified libraries (except the regular input library) due to the small insert size and the small total amount of total library after amplification. The first library was subjected to 101-cycle paired-end sequencing (one lane) and the second library was subjected to 101-cycle single-end sequencing (one lane) on the Illumina HiSeq. For pol III ChIP-BS-Seq, one library was prepared with eluate from 10 technical replicate RPC155 ChIP assays carried out as previously^38^ (~10 ng; all replicates were from the same batch of cross-linked HeLa cells, grown to 75% confluence), following the Illumina ChIP-Seq library protocol with bisulfite conversion as for the first input library. The pol III library was subjected to 101-cycle single-end sequencing (three lanes) on the Illumina HiSeq.

### ChIP-BS-Seq analysis

A novoalign index was created for the hg18 genome (UCSC), plus the Lambda genome (Genbank accession number J02459.1) and adapter sequences (Illumina PE PCR Primer 2.0) with these options: novoindex -k 13 -s 3 -b. Reads were aligned with novoalign using paired-end (-FILMFQ -t120 -h120 -b2 -a -i PE 20–600) or single-end settings (-FILMFQ -t120 -h120 -b2 -a). Repeat alignments intersecting at least one uniquely aligned sequence realigned using single-end settings, allowing up to seven alignments (-r A 7), which includes 85% of the repeat reads. For input, we obtained 94122299 genomic alignments (86740371 uniquely aligned reads and 2491119 reads with 2–7 alignments); for RPC155, we obtained 43883712 genomic alignments (37007535 uniquely aligned reads and 2694788 reads with 2–7 alignments). In addition, 1344324 reads of the input library and 1187215 reads of the pol III library aligned to the Lambda genome. The rates of conversion of methylated cytosines in the reads aligning to Lambda are 99.87% and 99.64% for input and pol III libraries, respectively.

Analysis of aligned data was performed with the USeq package of programs (useq.sourceforce.net). For ChIP-Seq analysis, alignments were converted to single-position point data with NovoalignParser (with no quality filter, to include repeat alignments) and peaks were called with ScanSeqs (using peakshift of 116 bp, as determined by PeakShiftFinder, and 300 bp window) and EnrichedRegionMaker with thresholds of 20 or 70, 13 and 1 for *Q*-value false discovery rate (FDR), empirical FDR and log2 ratio, respectively. To obtain a list of enriched pol III-transcribed genes (including SINEs), pol III-enriched regions (*Q*-value 20 threshold) were intersected with an annotation file containing all SINEs, tRNA genes and fragments, and other pol III-transcribed genes and elements from the RepeatMasker track of UCSC. The annotated genes/repeat elements were scored by DefinedRegionScanSeqs using the PointData and *Q*-value 70 threshold was used. The number of enriched genes by class is 1,472 Alu, 242 tRNA, 83 MIR, seven U6, four hY, three 7SL/SRP, three HVG, two 5S rRNA, two miRNA, one RNaseP, one MRP, one BC200 and one 7SK to a total of 1,822 genes.

For methylation analysis, alignments were converted to per-base methylated and unmethylated cytosines with NovoalignBisulfitParser (with no quality filter to include repeat alignments). Regions statistically enriched or reduced for methylation were determined with BisSeq (using settings -w 10 -m 10 -f 13 -l 0). Percent methylation scores for enriched pol III target genes (including SINEs) were obtained using ScoreMethylatedRegions.

To compare pol III enrichment scores and methylation fractions between Alu families (for example, AluJ, AluS and AluY), a non-parametric Kruskal–Wallis test was performed, followed by Dunn’s multiple comparison test.

For alignment of reads to the Alu consensus sequences, Alu sequences were obtained from Repbase and concatenated with 100xN separating the sequences. This fasta file was indexed with novoindex. Genomic reads were filtered for those intersecting with SINEs, converted to fastq format and realigned to the Alu consensus index with novoalign (-r All 14 -t240 -h120 -b2 -a -s). Per-base cytosine methylation graphs were obtained with BisStat and visualized with IGB (www.bioviz.org).

### Affinity chromatography

Separation of genomic DNA according to CpG methylation status was achieved by affinity chromatography with immobilized recombinant MBD2b and MBD3L1 using a MethylCollector Ultra (Active Motif), according to the manufacturer’s specifications.

### Gene expression analysis

To synthesize complementary DNA for RT–PCR, SuperScript III reverse transcriptase (Invitrogen) was used as previously^61^, with 200 ng of RNA and Hexanucleotide Mix (Roche). Primers and amplification conditions are described in Supplementary Table 1. Primer extension was performed using 5-carboxyfluorescein end-labelled (Invitrogen) Alu 21mer primer. RNA (5–10 μg) was denatured with 100 ng of labelled probe in 20 μl of 1 × First Strand Buffer (Invitrogen) at 80 °C for 10 min. Primer annealing was performed at 56 °C for 2 h. Thirty microlitre of elongation mix containing 100U of SuperScript III (Invitrogen), 1:50 RNAsin (Promega), 2 mM dithiothreitol, 1 mM dNTP and 10 ng μl^−1^ actinomycin D (Sigma) were added and samples were incubated at 42 °C for 1 h. Nucleic acids were precipitated in 0.1 M NaOAc and ethanol overnight with 1 μl of 1 M purified yeast tRNA as carrier. Samples were resolved on 7% sequencing gels and visualized using Typhoon 9400 (GE Healthcare). All *P* values were obtained using a two-tailed unpaired *t*-test.

### Translocation assay

Culture of Hom Alu mouse ES cells and translocation assays were conducted as previously^40^. Cells (10^7^) were electroporated with 25 μg of pTK-hyg or pCBAS and were allowed to recover from electroporation for 10 h before treatment for 16 h with 5-azacytidine or bleomycin. Cells were then allowed to recover in growth medium for 24 h and then subjected to selection with 200 μg ml^−1^ neomycin for 8–10 days. A plate without selection was used to calculate the loss of cell viability due to drug treatment. Colonies obtained after selection were GIEMSA stained for counting or picked for PCR analysis.

## Author contributions

D.V., J.V-A. and A.J.O. carried out the experiments and data analysis. V.H.C. provided the advice, lab space and reagents to allow D.V. to address referee’s comments. B.R.C. and A.J.O. designed and interpreted the ChIP-BS-Seq analysis. R.J.W. conceived the study, supervised D.V. and J.V-A. and wrote the manuscript.

## Additional information

**Accession codes:** ChIP-BS-Seq data deposited in the NCBI Gene Expression Omnibus database under GEO accession number GSE38794.

**How to cite this article:** Varshney, D. *et al*. SINE transcription by RNA polymerase III is suppressed by histone methylation but not DNA methylation. *Nat. Commun.* 6:6569 doi: 10.1038/ncomms7569 (2015).

## Supplementary Material

Supplementary Figures, Tables and References Supplementary Figures 1-19, Supplementary Table 1 and Supplementary References

Supplementary Dataset 1 Dataset for pol III ChIP-Seq and ChIP-BS-Seq analyses. A key is provided in the description sheet.

Supplementary Dataset 2 Details of individual experiments testing how Alu translocation assays respond to 5-azacytidine and bleomycin. Students t-test was used to calculate the p-values for three independent biological replicates.
