# Supplementary material for: SINE transcription by RNA polymerase III is suppressed by histone methylation but not by DNA methylation
Source: Nat Commun. 2015 Mar 23;6:6569. doi: 10.1038/ncomms7569 (PMC4382998; doi:10.1038/ncomms7569)
Supplement: Supplementary Figures, Tables and References — Supplementary Figures 1-19, Supplementary Table 1 and Supplementary References [file ncomms7569-s1.pdf]

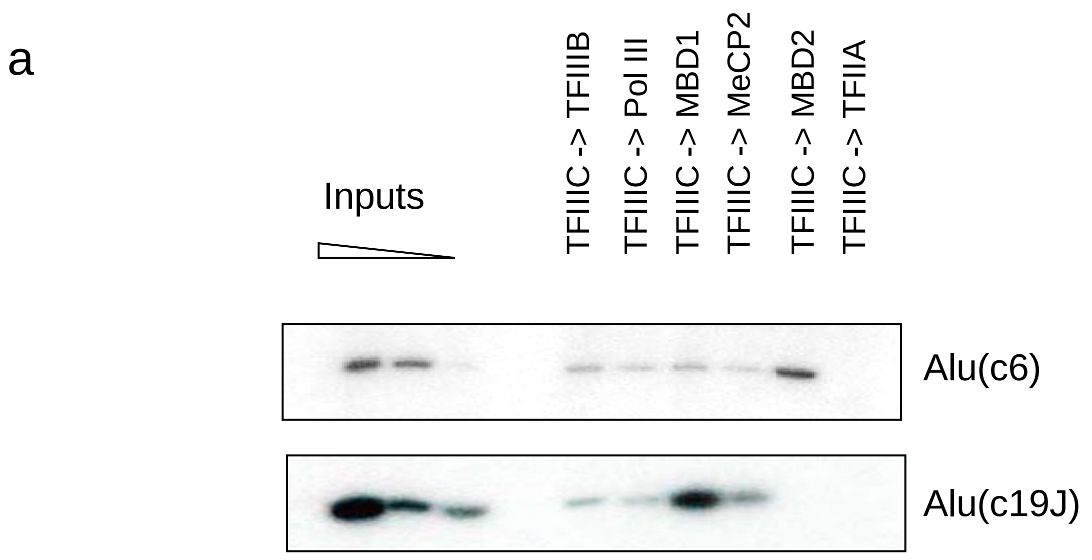

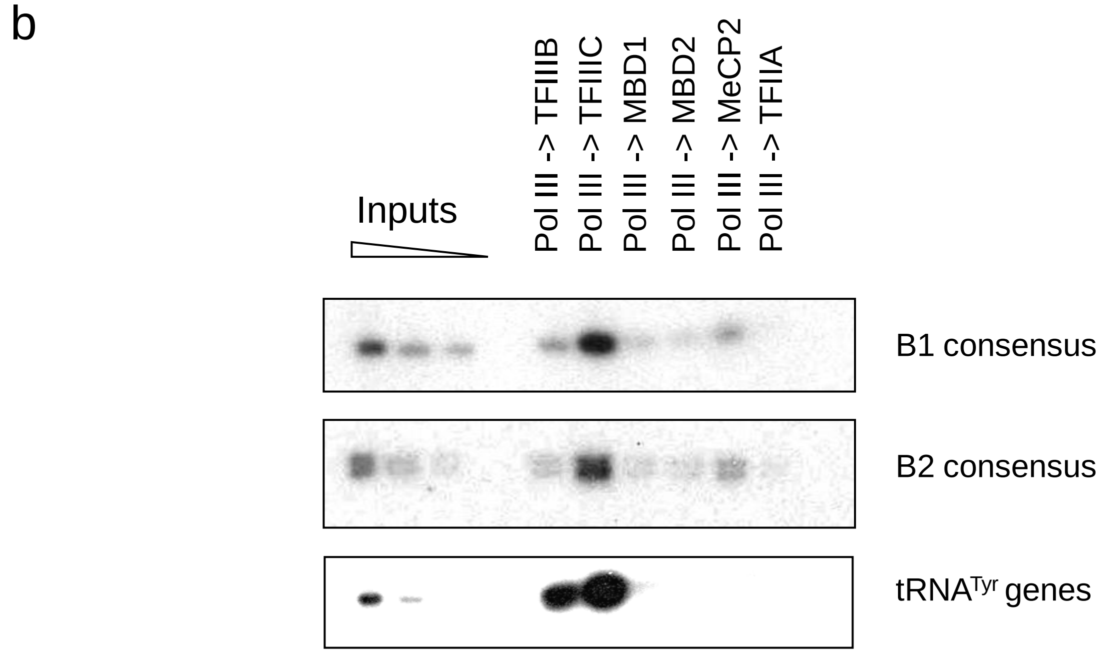

Supplementary Figure 1 Pol III and TFIIIC co-occupy SINEs with MeCP2, MBD1 and MBD2. (**a**) Sequential semi-quantitative ChIP in which HeLa cell DNA immunoprecipitated using TFIIIC antibody was re-precipitated using antibodies against pol III, TFIIIB, TFIIA (negative control), MBD1, MBD2 and MeCP2, as indicated, and then assayed with primers that amplify Alu(c6) or Alu(c19J) loci specifically. (**b**) Sequential semi-quantitative ChIP in which A31 fibroblast DNA immunoprecipitated using pol III antibody was re-precipitated using antibodies against TFIIIC, TFIIIB, TFIIA (negative control), MBD1, MBD2 and MeCP2, as indicated, and then assayed with primers that amplify B1 or B2 consensus sequences or $tRNA^{Tyr}$ genes.

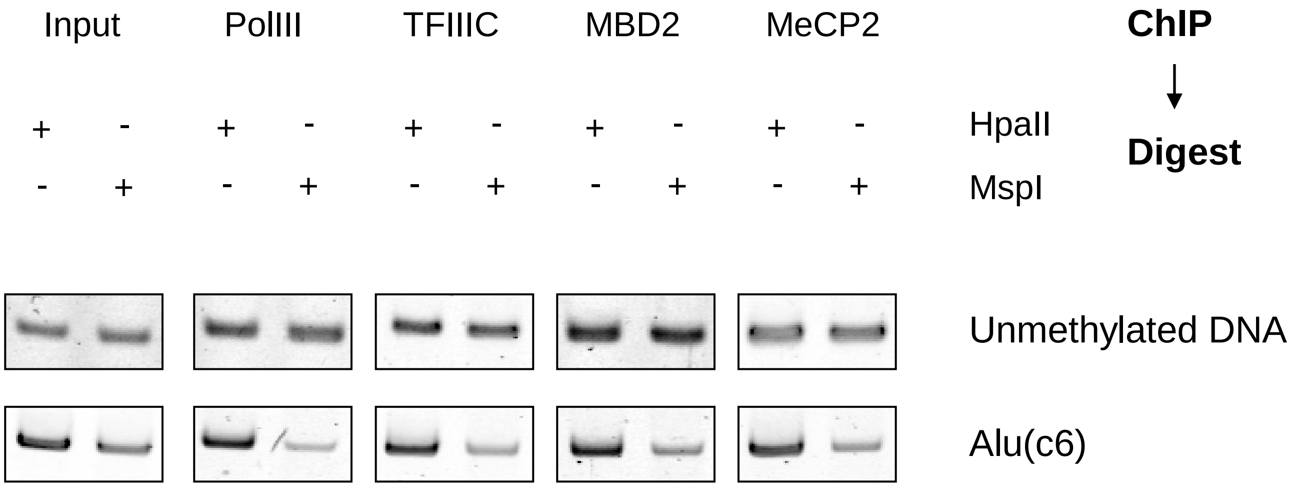

Supplementary Figure 2 ChIP-Chop assay demonstrating methylation of genomic DNA bound by pol III, TFIIIC, MBD2 and MeCP2. Genomic DNA isolated from HeLa cells by ChIP with antibodies to pol III, TFIIIC, MBD2 and MeCP2 was digested with HpaII or Msp1, as indicated, before PCR amplification with specific primers to Alu(c6). ChIP DNA was spiked prior to digestion with unmethylated PCR product containing a HpaII/MspI site, which was then used to normalise for digestion efficiency.

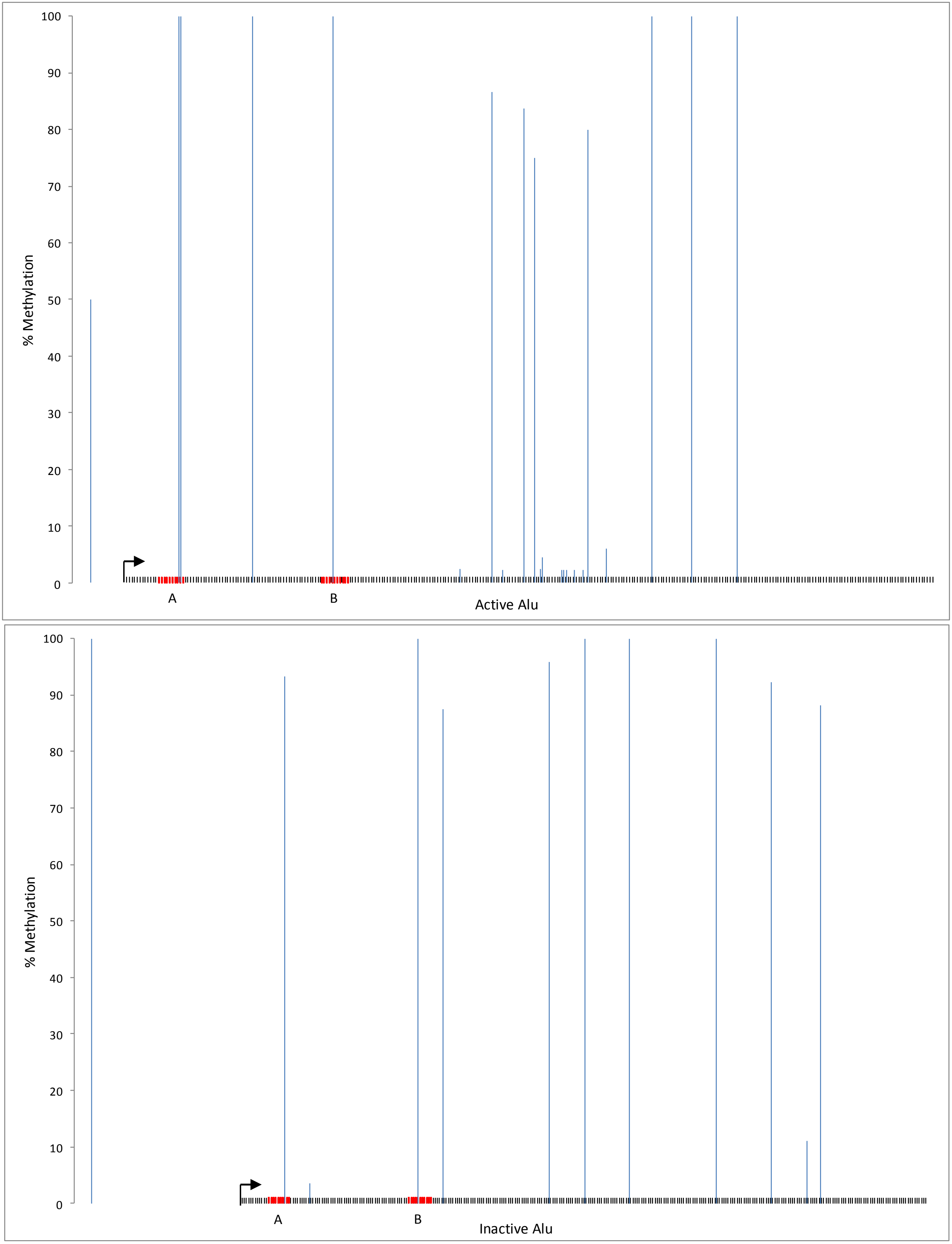

Supplementary Figure 3 Comparison of the extent of methylation in HeLa cells of two individual Alu loci, as determined by bisulfite sequencing. The height of a vertical blue line indicates % methylation at a particular C, whereas the short black vertical lines indicate each bp of the Alu. Arrowhead indicates transcription start site and direction. Upper panel represents an Alu on chromosome 10 (hg19 coordinates 94035667-94035971) that is occupied by pol III in HeLa cells, whereas lower panel represents an Alu on chromosome 11 (hg19 coordinates 48002316-48002625) that shows minimal pol III binding in the same cells. A- and B-block promoter elements are indicated.

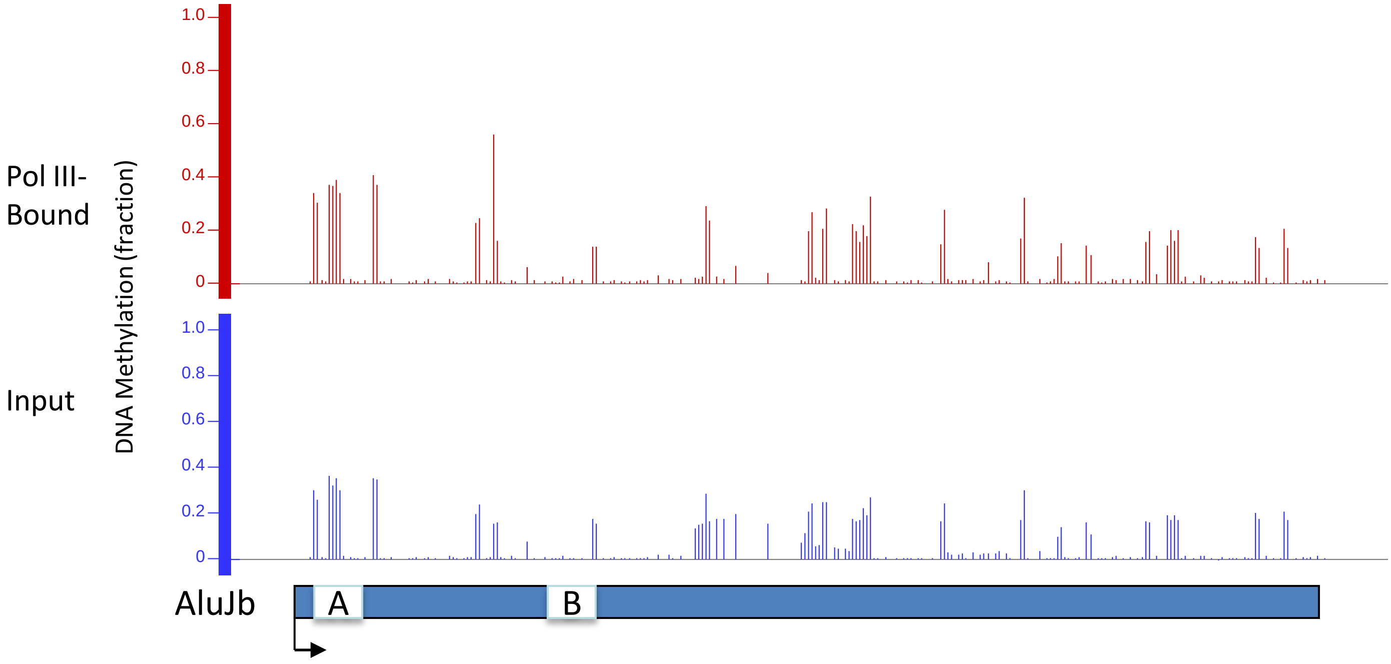

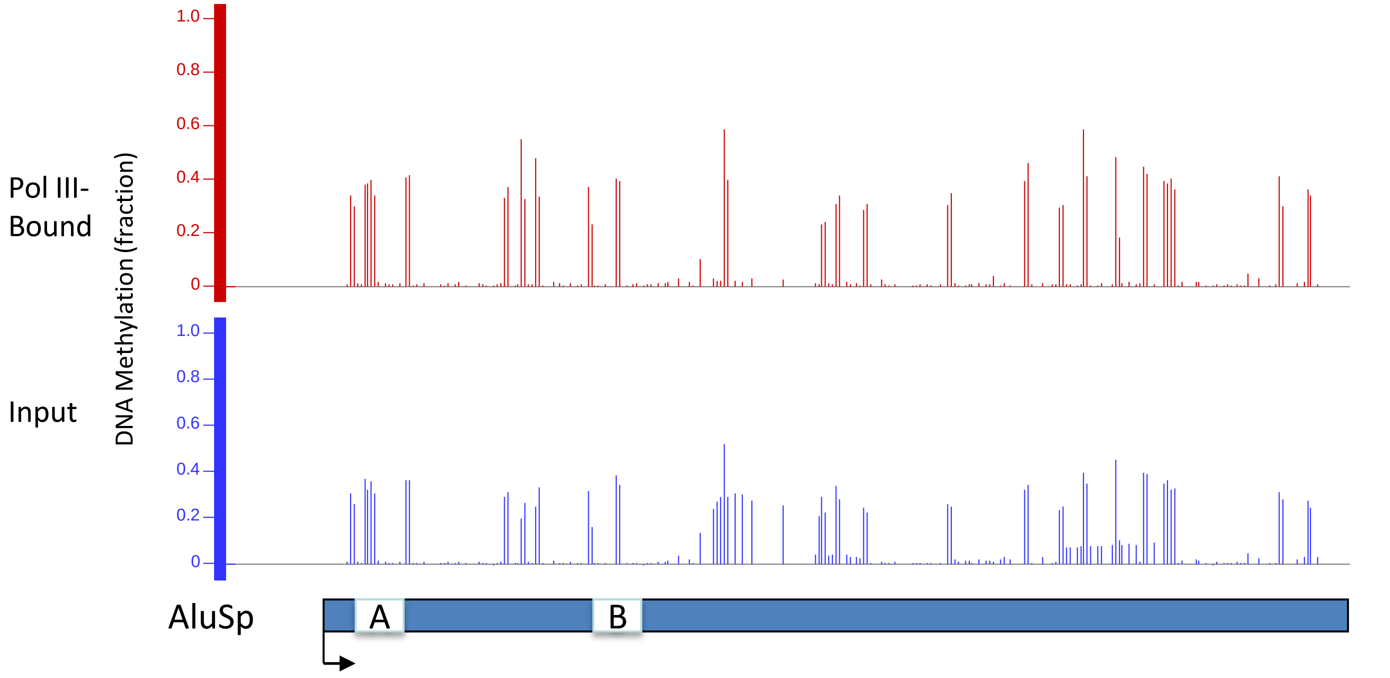

Supplementary Figure 4 Comparison of the extent of methylation of the AluJb (**a**) and AluSp (**b**) subfamilies between genomic DNA (input) and pol III-bound DNA, as determined by ChIP-BS-Seq. The height of a vertical line indicates fraction of methylation at a particular C within the subfamily consensus sequence. A- and B-block promoter elements are indicated.

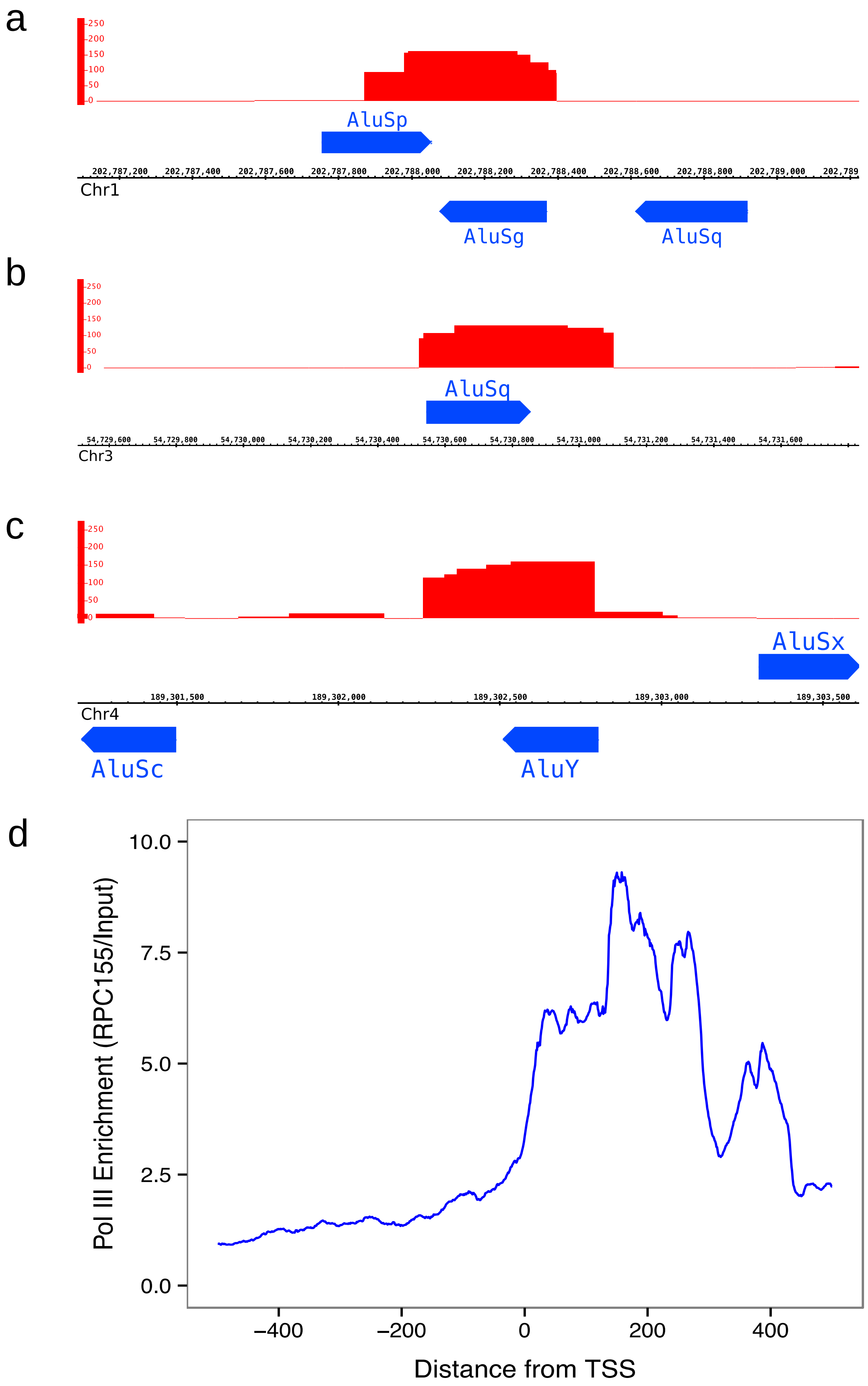

Supplementary Figure 5 Pol III has access to the entire length of some Alu SINEs. (**a-c**) Screenshots of pol III binding, as revealed by ChIP at three chromosomal loci (coordinates indicated) containing members of the indicated Alu families. Locations of Alu sequences are indicated in blue (blunt end denotes start). Sites of pol III detection are indicated in red, with y-axis representing extent of occupancy. Pol III occupancy can be seen to be selective for particular Alus. (**d**) Class average distribution of pol III relative to transcription start site (TSS) at occupied Alu SINEs. Although the Alu consensus sequence is 282bp long, a peak of pol III is detected further downstream, suggesting significant readthrough into flanking DNA.

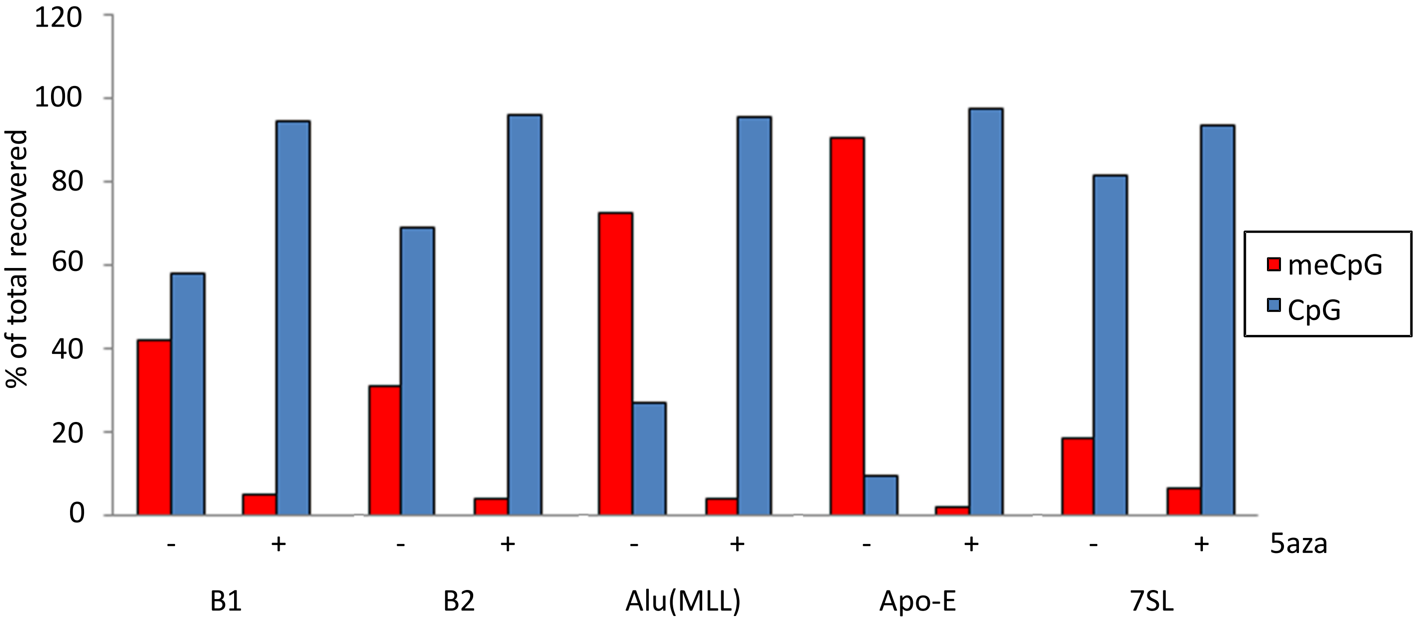

Supplementary Figure 6 Treatment of cells with 5-azacytidine causes extensive demethylation of genomic DNA. A MethylCollector™ Ultra column was used to separate into methylated and unmethylated fractions genomic DNA from mouse ES cells that were mock treated (-) or treated (+) for 16 hrs with 5-azacytidine. Sequences were then amplified using primers to B1 and B2 consensus sequences, the Alu(MLL) locus and Apo-E and 7SL genes.

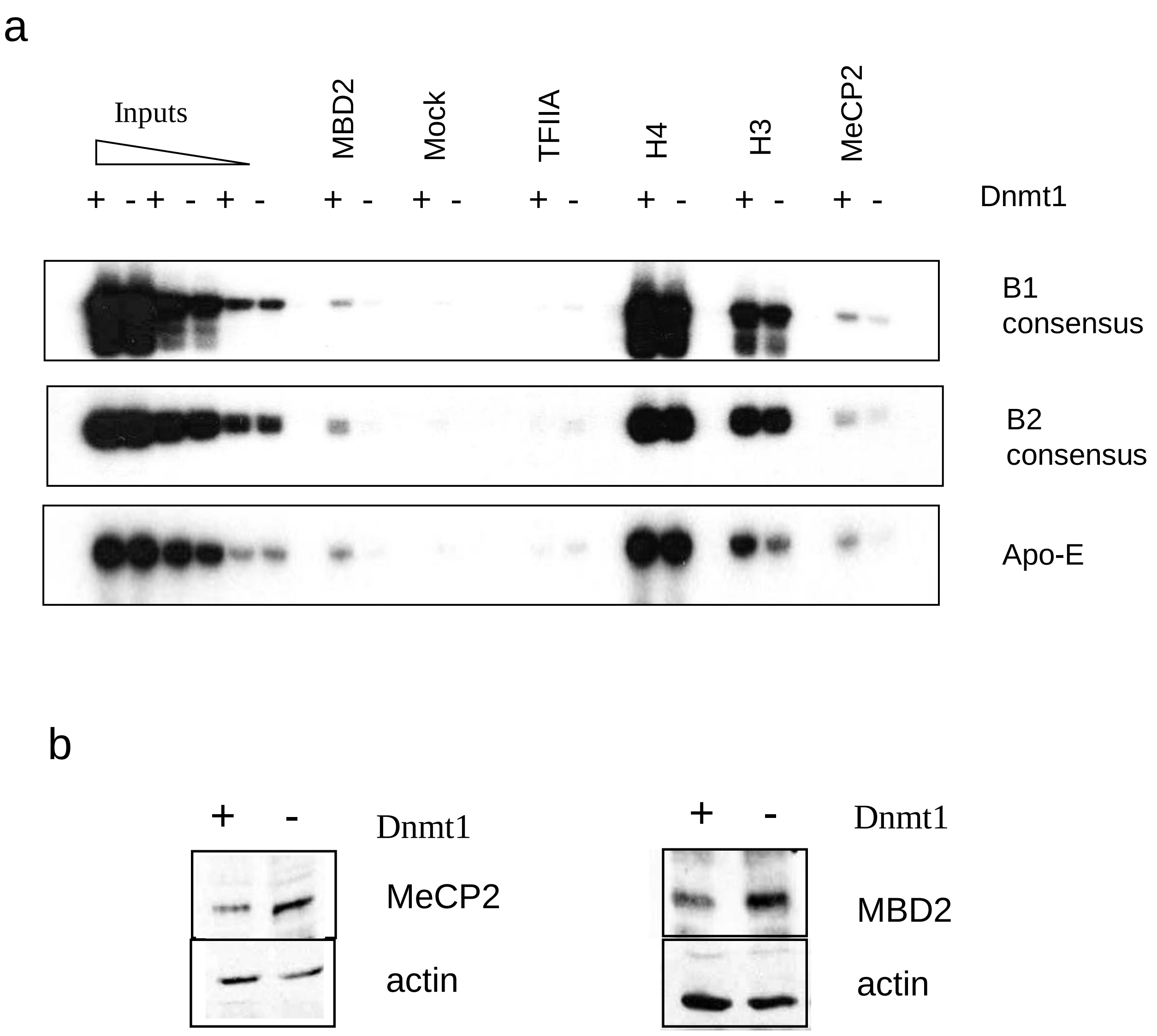

Supplementary Figure 7 Binding of MBPs to SINEs is methylation-sensitive. (**a**) ChIP assay in matched Dnmt1$^{+/+}$ and Dnmt1$^{-/-}$ fibroblasts showing occupancy of MBD2 and MeCP2 at B1 and B2 loci, as well as the Apo-E gene. ChIPs for TFIIA and without antibody (mock) provide negative controls. Positive controls are provided by antibodies against histone H4 and histone H3. (**b**) Western blots confirming presence of MBD2 and MeCP2 in Dnmt1$^{-/-}$ fibroblasts. Actin provides a loading control. The elevated MBP signal from Dnmt1$^{-/-}$ fibroblasts, relative to Dnmt1$^{+/+}$, is thought to reflect improved recovery from chromatin when DNA is hypomethylated.

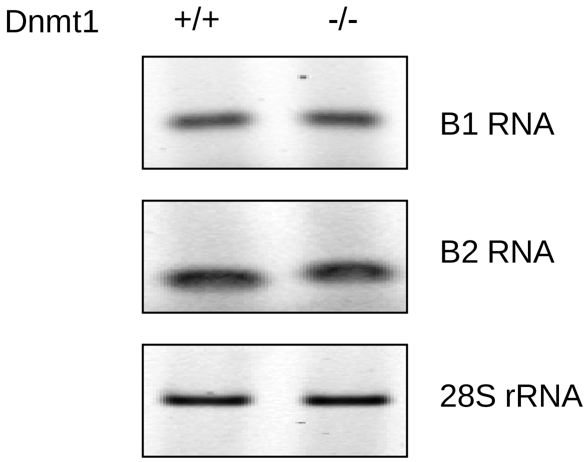

Supplementary Figure 8 Pol III-dependent expression of B1 and B2 SINEs is not elevated by knockout of Dnmt1. Analysis by RT-PCR of expression levels of the indicated transcripts in matched Dnmt1$^{+/+}$ and Dnmt1$^{-/-}$ fibroblasts cultured in the presence of 50µg/ml α-amanitin for 24hrs. 28S rRNA provides a loading control.

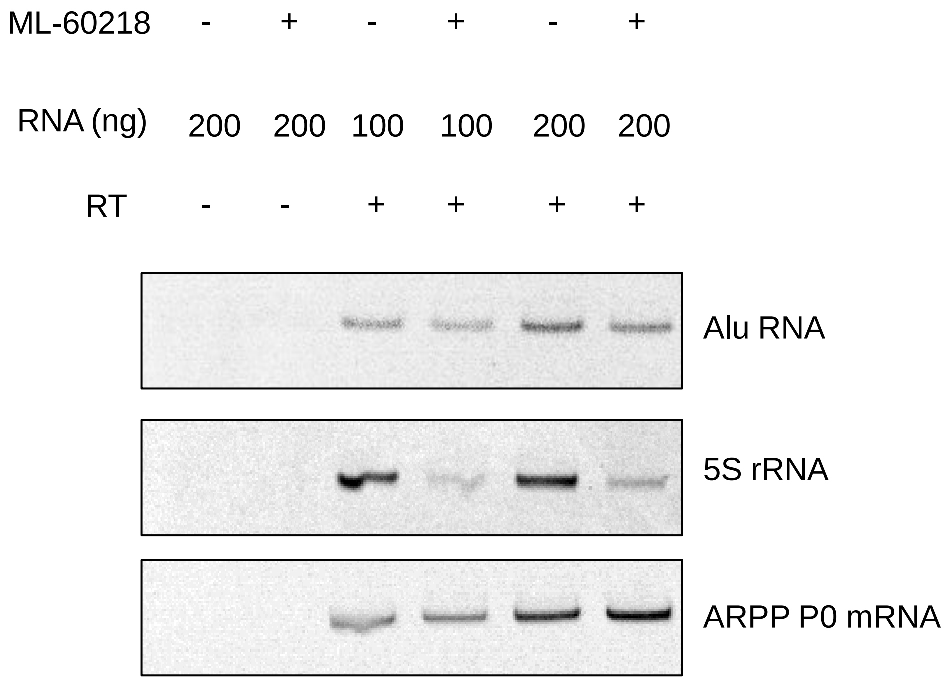

Supplementary Figure 9 The pol III-specific inhibitor ML-60218 decreases expression of Alu RNA detected by RT-PCR. HeLa cells were treated for 24hr with or without 20μM ML-60218 (Symansis) and expression of the indicated transcripts was assayed by RT-PCR using 100ng or 200ng of RNA as template. Reverse transcriptase was omitted in lanes 1 and 2. 5S rRNA expression is pol III-dependent and serves as a positive control for efficacy of ML-60218, whereas ARPP P0 mRNA is pol III-independent and serves as control for specificity. Because both pol II and pol III contribute to Alu expression, a partial response is seen that is less strong than the decrease in 5S rRNA. We conclude that the assay is sensitive to changes in Alu transcription by pol III and that Alu RNA generated by pol II does not mask changes in the pol III output.

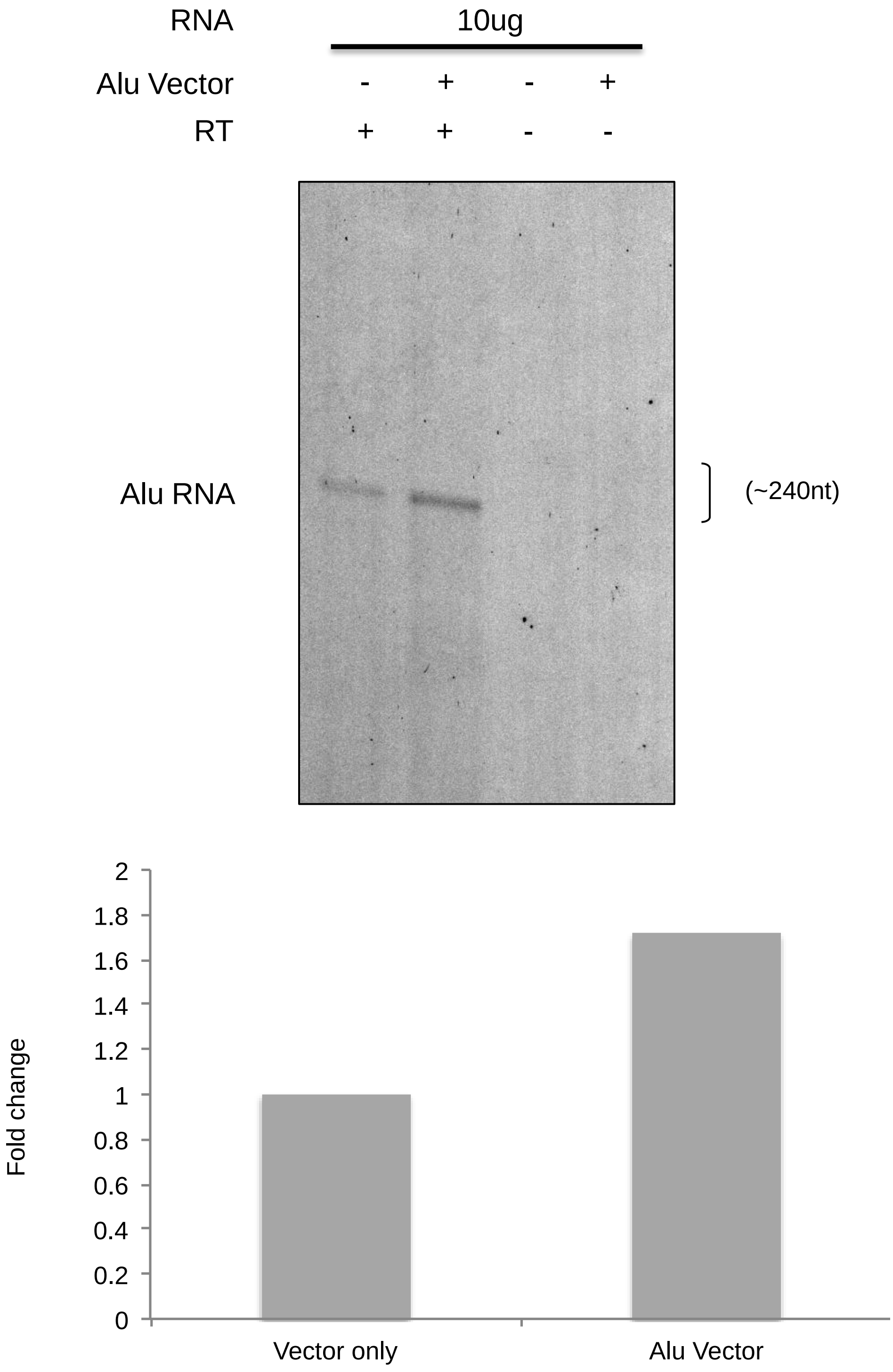

Supplementary Figure 10 The primer extension assay specifically detects pol III-transcribed Alu transcripts. Primer extension with Alu21mer analysing the expression levels of Alu transcripts initiated from the pol III start site in HeLa cells transfected with empty vector and vector containing an Alu element. The Alu vector was created by amplifying a specific Alu locus by PCR using Chr1AluY primers, followed by TA cloning into a pGEM® T-easy vector (Promega) which lacks mammalian promoters. Reverse transcriptase was omitted from the reactions in lanes 3-4. Even loading was confirmed by ethidium bromide staining. Lower panel shows the quantification of the primer extension product by ImageJ. The data show the mean fold change of two independent biological replicates.

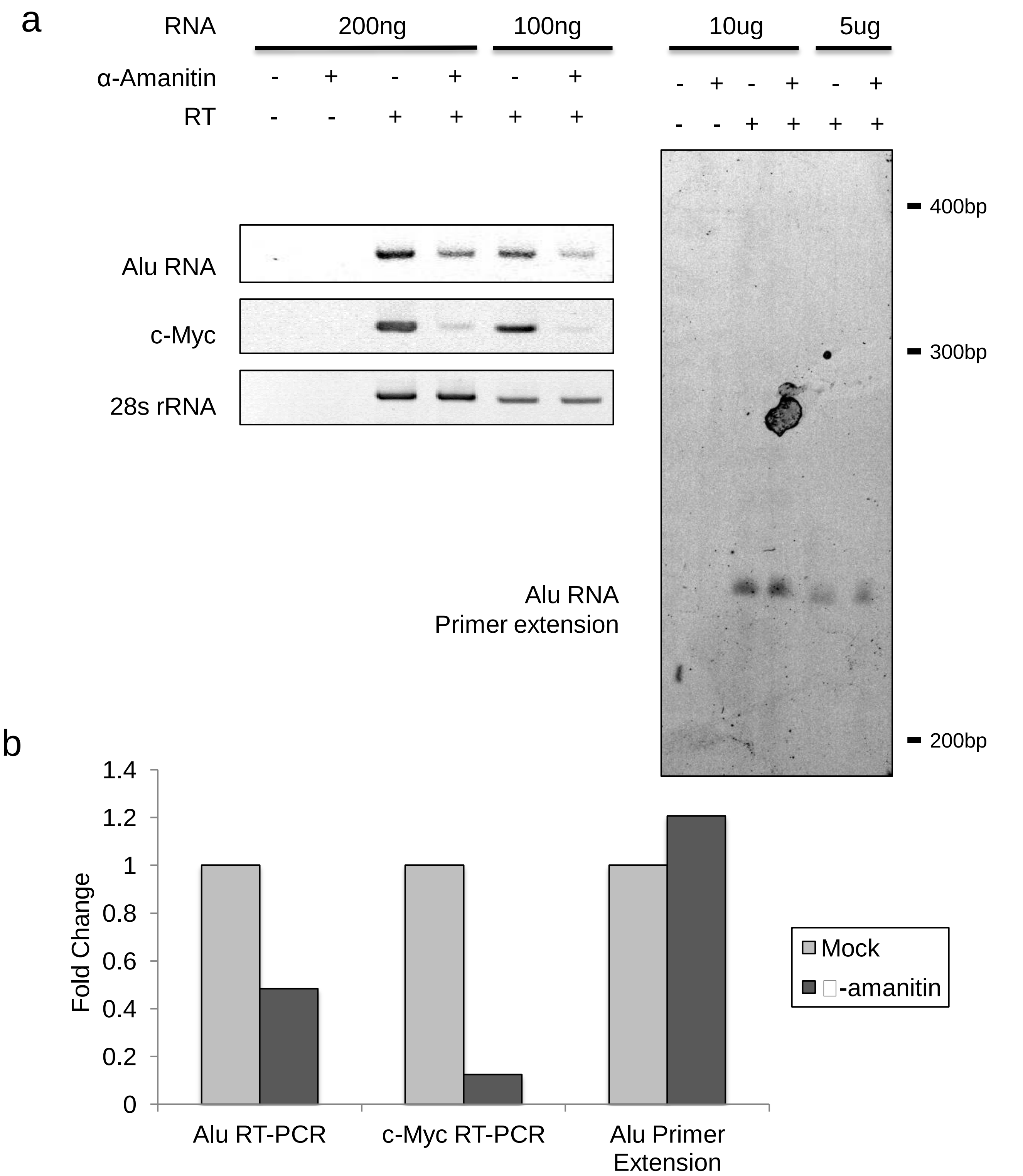

Supplementary Figure 11 Alu transcripts initiated from the pol III start sites are resistant to 50μg/ml α-amanitin. **a)** Analysis by RT-PCR of expression levels of the indicated transcripts in HeLa cells cultured in the presence of 50μg/ml α-amanitin for 8hrs. Pol I-derived 28S rRNA provides an α-amanitin-resistant loading control, whereas pol II-derived c-Myc mRNA provides a control for the efficacy of pol II inhibition by α-amanitin. The panel on the right shows primer extension using the Alu21mer primer assaying expression levels of transcripts initiated from the pol III start site of the AluYa5 family, to give a single ~240bp extension product. Of the ~3,918 AluYa5 family members, the one that is closest to an annotated pol II start site would give a ~350bp product in this assay, were it expressed by pol II. Reverse transcriptase was omitted from the reactions in lanes 1-2. **b)** Fold change in Alu and c-Myc expression quantified using ImageJ. RT-PCR signals were normalised to 28S rRNA. The Alu transcripts detected by this primer extension assay are not inhibited by 50μg/ml α-amanitin because they are generated by pol III; in contrast, Alu expression detected by RT-PCR includes pol II-derived read-through transcripts and is therefore reduced by 50μg/ml α-amanitin.

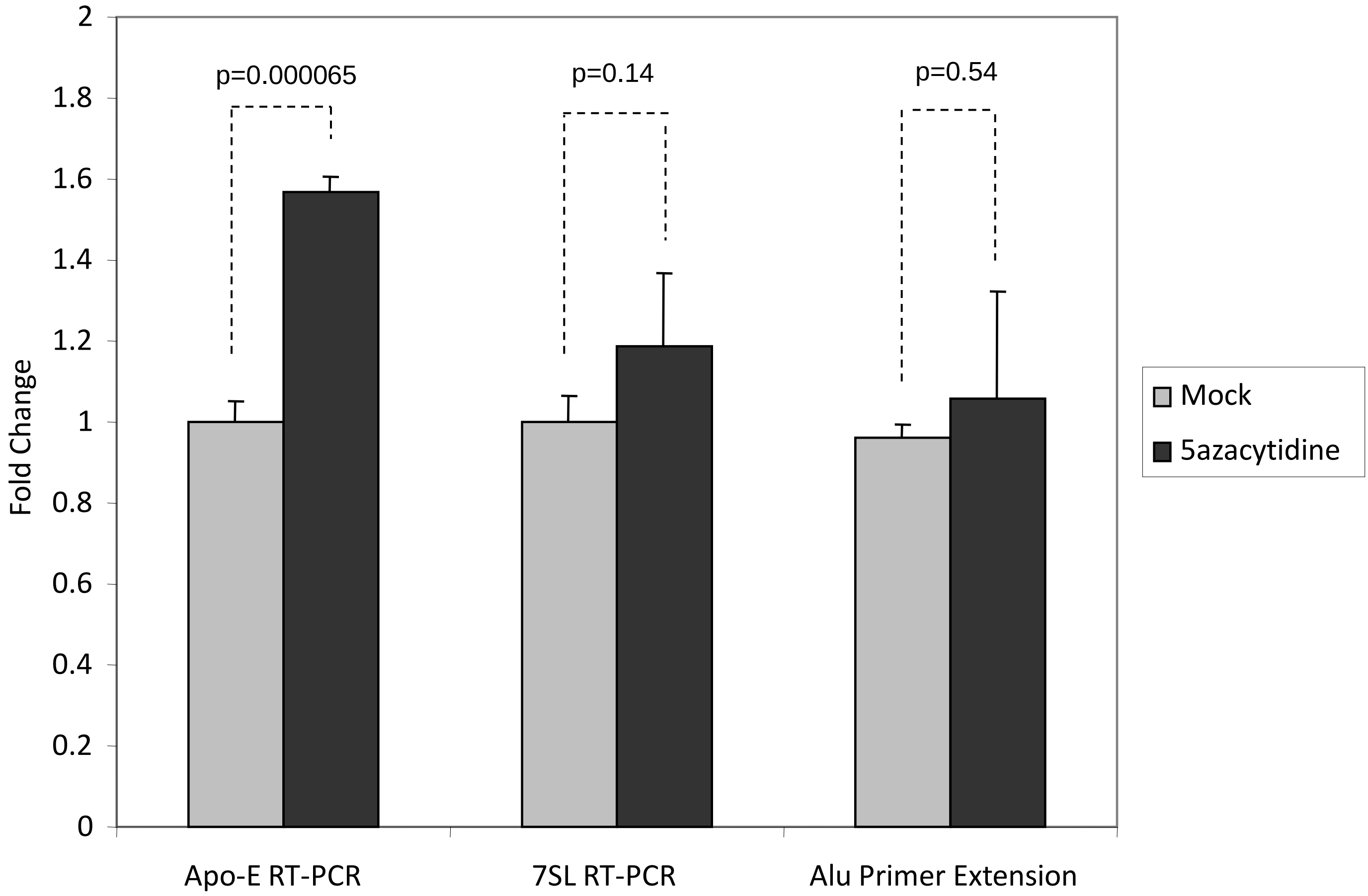

Supplementary Figure 12 5-azacytidine treatment has little effect on expression of Alu transcripts initiated from the pol III transcription start site. Mean +/- standard deviation of the fold change in Apo-E mRNA, 7SL RNA and Alu RNA, after normalization to ARPP P0 mRNA loading control, in HeLa cells treated for 72 hrs with 5-azacytidine. Apo-E and 7SL were assayed by RT-PCR, whereas Alu RNA was analysed by primer extension, to specify transcripts initiated at the pol III start site, and quantified using ImageJ. Whereas Apo-E mRNA displays a significant increase in response to 5-azacytidine (p=0.000065, t-test), this is not the case for 7SL (p=0.14, t-test) or Alu RNA (p=0.54, t-test). n=3.

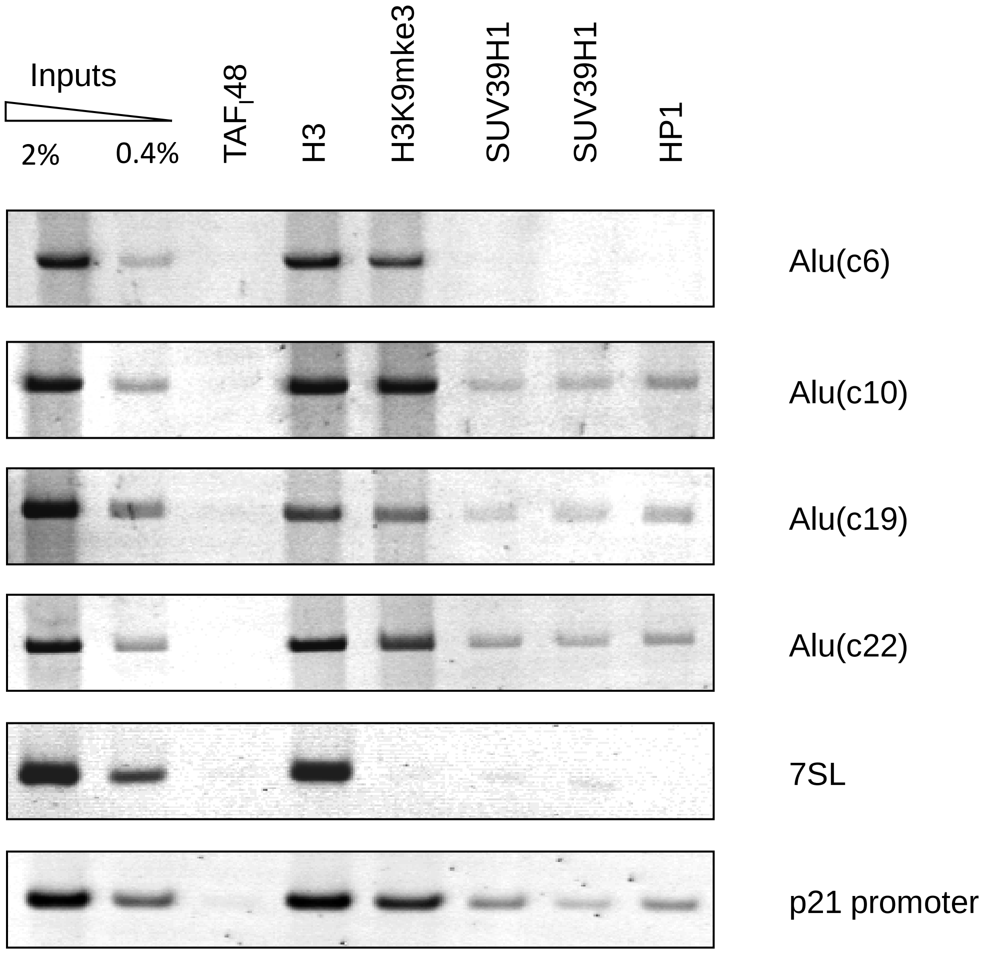

Supplementary Figure 13 SUV39H1 associates specifically with some Alu loci. ChIP assay in HeLa cells showing crosslinking of H3K9me3, SUV39H1 and HP1 at Alu loci from chromosomes 6, 10, 19 and 22, as well as 7SL and p21 (positive control) genes. ChIPs for histone H3 and TAF$_I$48 provide positive and negative controls, respectively. The two SUV39H1 lanes show ChIPs carried out with alternative SUV39H1 antibodies.

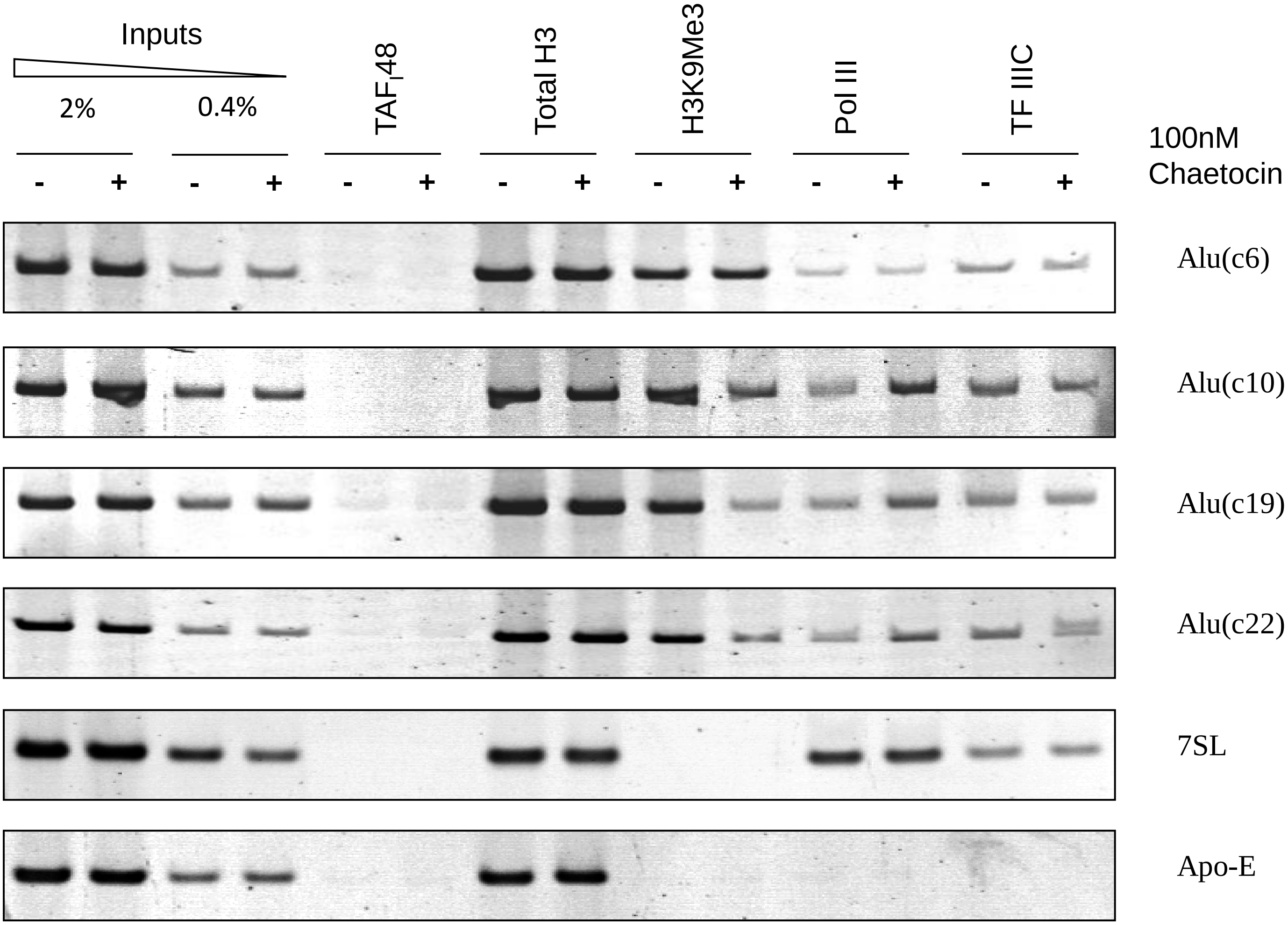

Supplementary Figure 14 Chaetocin treatment leads to elevated pol III binding at Alus. ChIP assay in HeLa cells treated for 24 hrs with vehicle (-) or 100nM chaetocin (+), showing binding of histone H3, TAF$_I$48, H3K9me3, pol III and TFIIIC at Alu loci, 7SL and Apo-E genes.

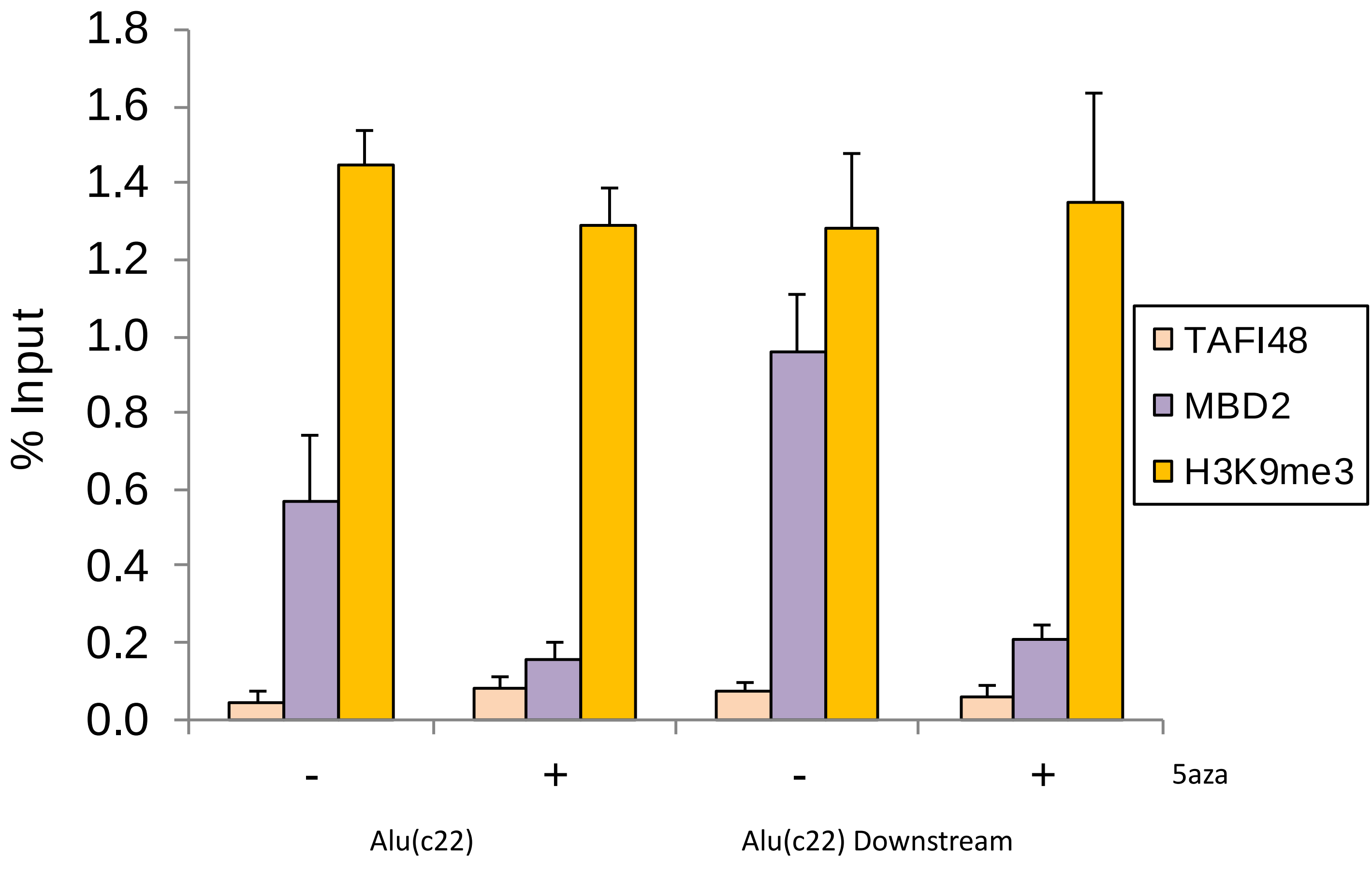

Supplementary Figure 15 5-azacytidine treatment does not reduce H3K9me3 occupancy at Alus. Mean +/- SEM of the percentage input bound in three independent ChIP-qPCR assays with HeLa cells treated for 72 hrs with (+) or without (-) 5-azacytidine, showing binding of TAF_I48, MBD2 and H3K9me3 to the body of Alu(c22) and its downstream region. The H3K9me3 data were normalized against total histone H3 to provide an indication of changes in the proportion of H3 with trimethylated K9. N.B. These data are from the same experiments as are shown in Fig 4b.

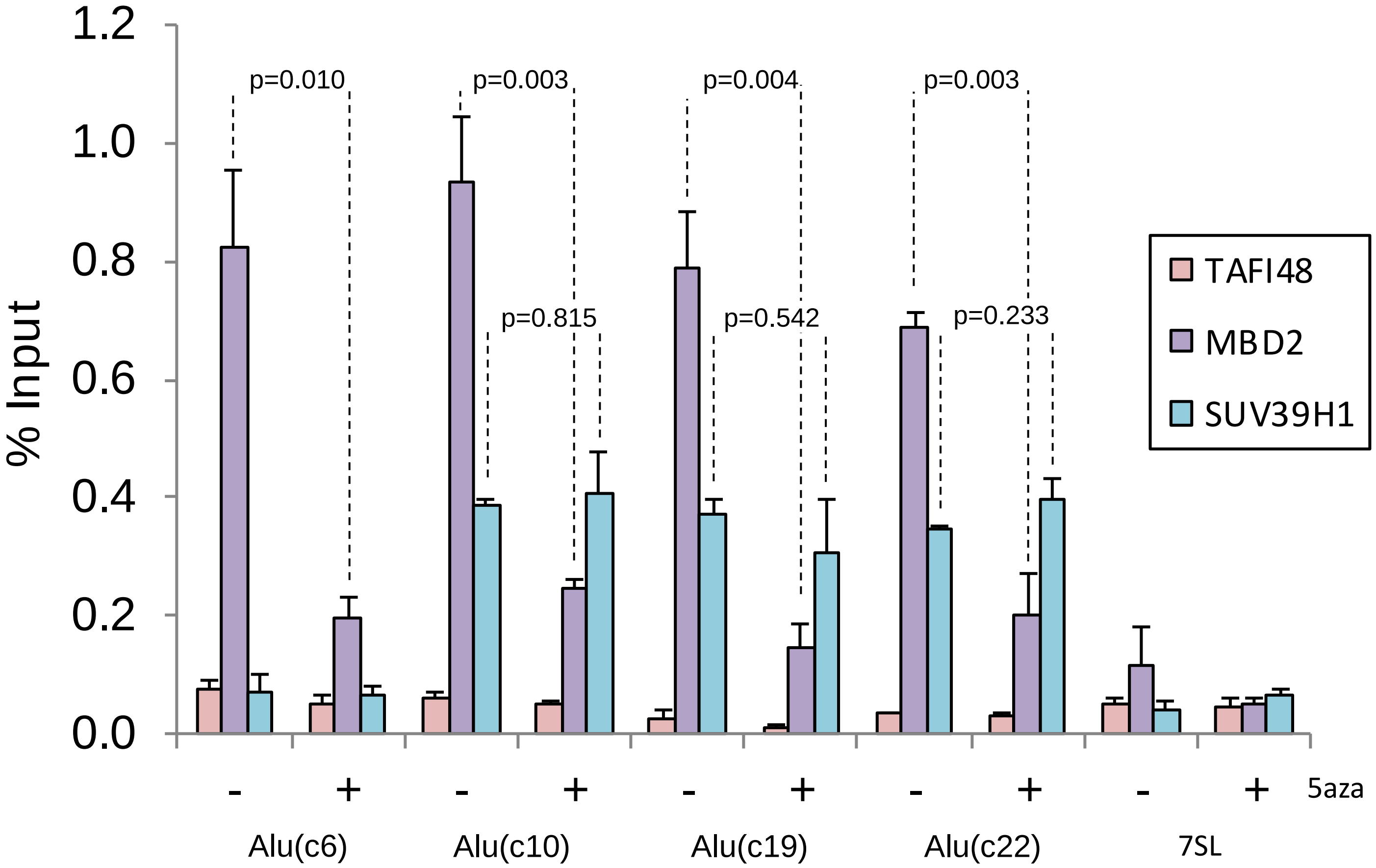

Supplementary Figure 16 5-azacytidine treatment does not reduce SUV39H1 occupancy at Alus. Mean +/- SEM of the percentage input bound in three independent ChIP-qPCR assays with HeLa cells treated with (+) or without (-) 5-azacytidine, showing binding of TAF_I48, MBD2 and SUV39H1 to 7SL and the indicated Alu loci. All p-values are calculated by t-test.

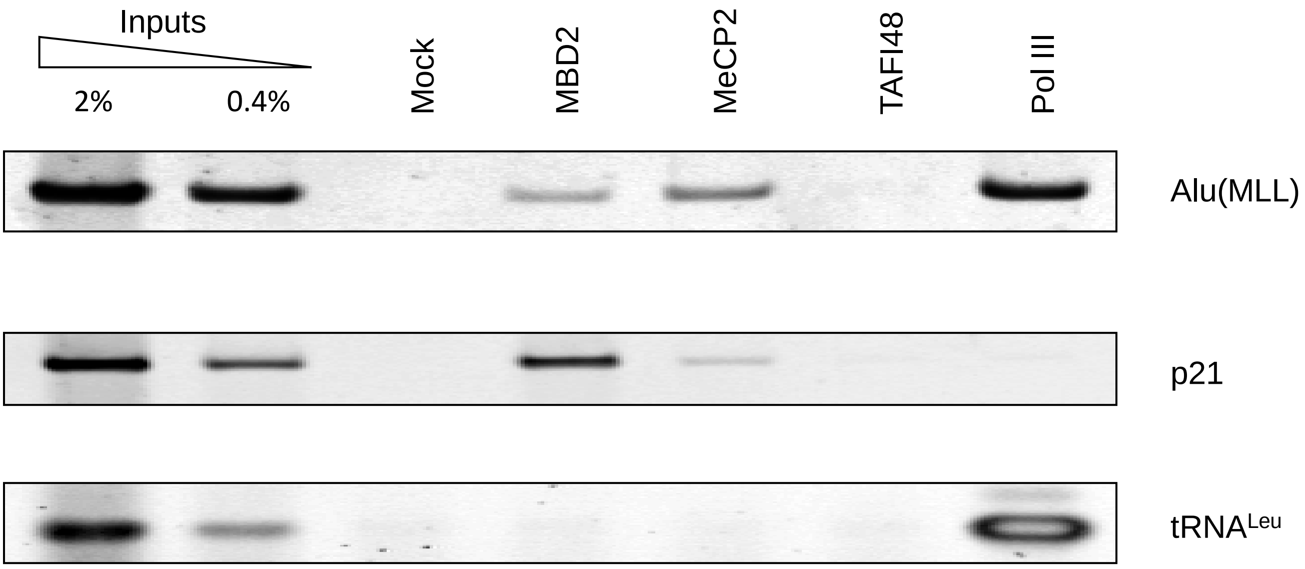

Supplementary Figure 17 MLL-derived Alu elements are occupied by pol III and MBPs in Hom Alu mouse ES cells. ChIP showing binding of pol III, MBD2 and MeCP2 to the human MLL gene-derived Alu in mouse cells. ChIPs for TAF_I48 and without antibody (mock) provide negative controls. The pol II-dependent p21 promoter provides a specificity control, showing occupancy by MBD2 and MeCP2, but not by pol III. In contrast, $tRNA^{Leu}$ genes show binding to pol III, but not to MBD2 or MeCP2.

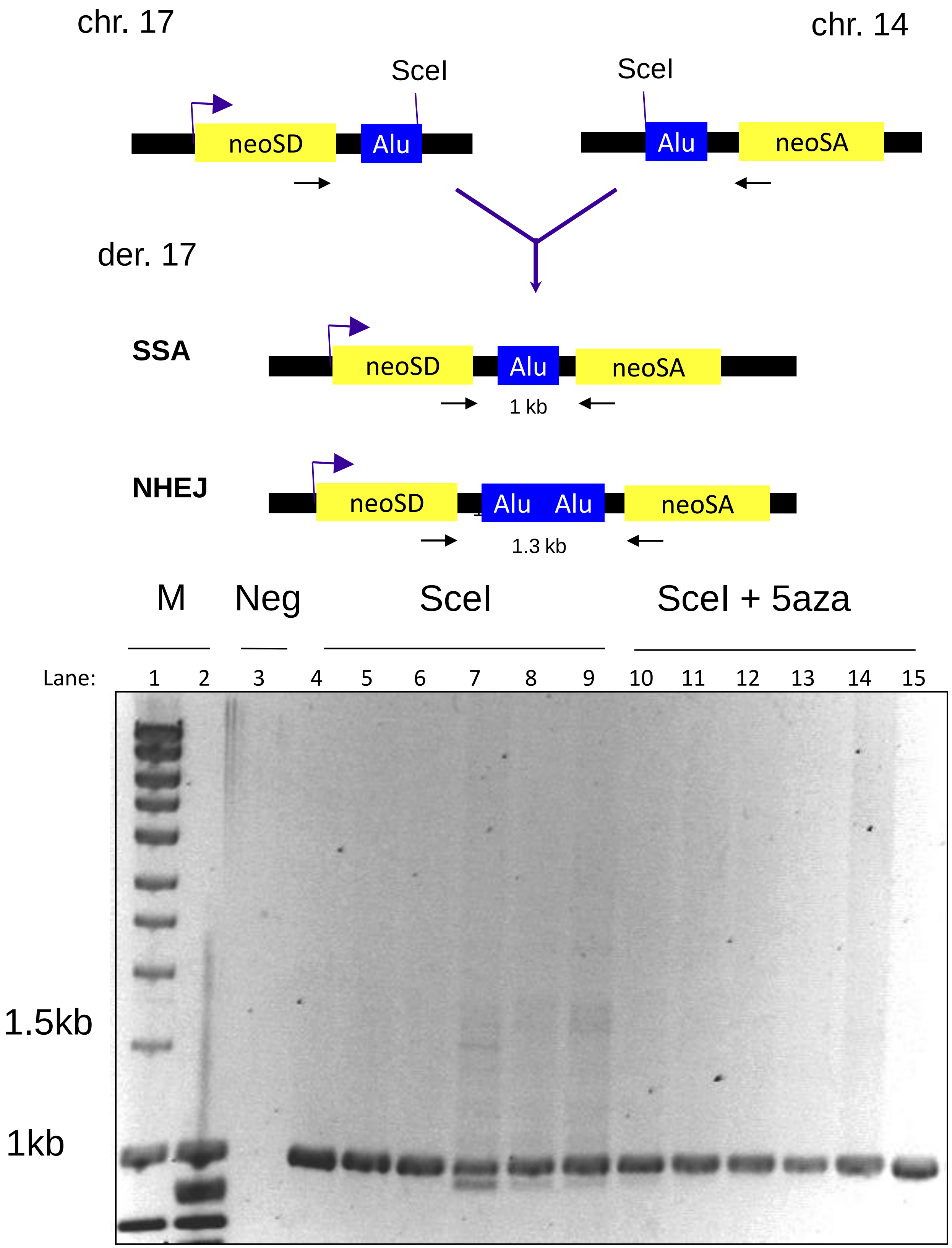

Supplementary Figure 18 Interchromosomal translocations between MLL-derived Alu elements are detected in neomycin-resistant colonies of Hom Alu ES cells following induction of a double-strand break with I-Sce1. A translocation-specific PCR product is detected using primers recognising the *neo* fragments from chromosomes 17 and 14. Rearrangements arising from single-strand annealing (SSA) are predicted to generate a 1kb PCR product, as observed in all cases; non-homologous end joining (NHEJ) would generate a 1.3kb product [37]. The negative control (Neg; lane 3) used the same cells without I-Sce1, which promotes translocation. Six separate translocation events (neomycin-resistant colonies) are shown from cells treated without (lanes 4-9) or with (lanes 10-15) 5-azacytidine. Lanes 1 and 2 contain molecular weight markers (M).

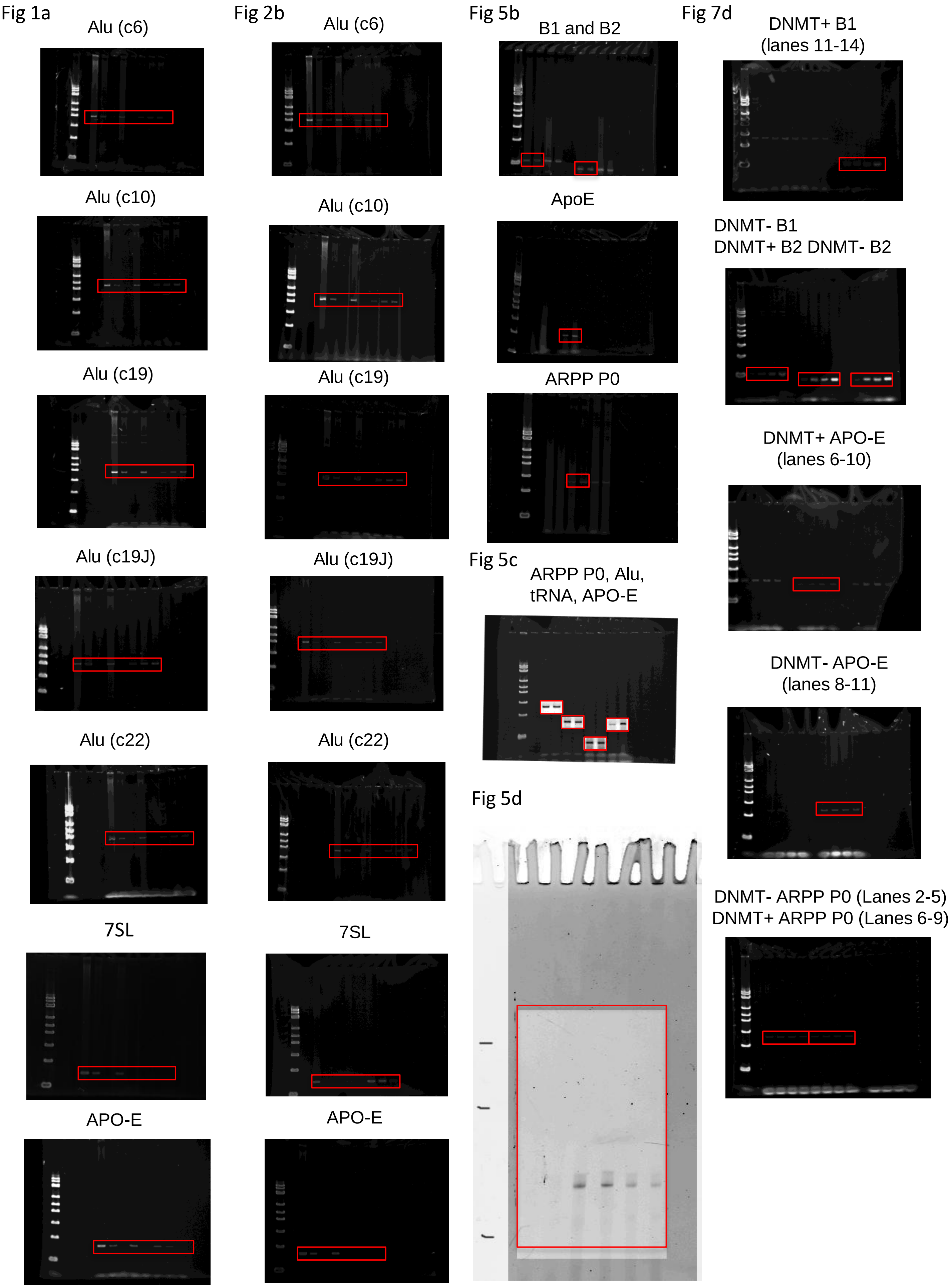

Supplementary Figure 19. Original uncropped gels of indicated figures. Red boxes highlight the bands shown in the figures.

Supplementary Table 1 Primers and amplification conditions. Forward (F) and reverse (R) primers are shown for amplification of the indicated locus in cDNA or DNA from human (h) and/or mouse (m) cells.

The following cycling parameters were used for PCR amplification, where T(a) and n are specified for each primer pair: 95°C for 30s, T(a) for 30s, 72°C for 30s for n cycles

[a] The forward primer hybridises to position 82-103 Alu PV consensus[1]. The reverse primer hybridises to position 238-222 Alu PV consensus[2]. The primer set amplifies 3918 anotated AluYA5 family members in the genomic assembly, only 42 of which lie within known or predicted pol II genes.

[b] The primer hybridises to position 238-218 Alu PV consensus sequence[2]. The primer hybridises to AluYA5 RNA and produces a 240bp Alu RNA specific extension product. A 350bp pol II-dependent product seen by Liu et al was not observed in our experiments.

[c] Primers amplify chr22:27011261-27011095, that is 207bp to 373bp downstream of Alu (c22). Co-ordinates refer to the UCSC hg19 assembly.

[d] The forward primer hybridises to position 8-27 B1 consensus sequence and the reverse primer hybridises to position 116-95 B1 consensus sequence. Both primers were obtained from[3].

[e] The forward primer hybridises to position 1-19 B2 consensus sequence and the reverse primer hybridises to position 84-67 B2 consensus sequence[4].

Locus name [Species]		Primers	T(a)	n
28S rRNA [h]	F	GCACGAGACCGATAGTCAAC	58 °C	12-15
	R	GGAGGACGGACGGACGGAC		
28S rRNA [m]	F	CCCGACGTACGCAGTTTTAT	58 °C	23-25
	R	CCTTTTCTGGGGTCTGATGA		
5S rRNA [h, m]	F	GGCCATACCACCCTGAACGC	58 °C	16-18
	R	CAGCACCCGGTATTCCCAGG		
7SL [h, m]	F	GTGTCCGCACTAAGTTCGGCATCAATATGG	70 °C	25-30
	R	TATTCACAGGCGCGATCCCACTACTGATC		
Alu consensus[a] [h]	F	ACCATCCCGGCTAAAACGGTGA	58°C	25-30
	R	GCGATCTCGGCTCACTG		
Alu 21mer[b] [h]	F	GCGATCTCGGCTCACTGCAAG	56°C	Primer ext.
Chr1AluY [h]	F	TGGCCATGTATAAGAACTAGGG	58°C	25-30
	R	TCCGATTTATTTGAGCCCTTA		
Alu (c6) [h]	F	CCAGAAAAATTACCAATTAGTTC	53 °C	25-30
	R	GGGCCTATTGACTATGCTTAC		
Alu (c10) [h]	F	GATTCTCAACAGCAGAATTCCA	53 °C	25-30
	R	CATGTTTGAGAATGTCTACTTC		
Alu (c19) [h]	F	CCACGTGTTTATCTGTAAGGTG	53 °C	25-30
	R	GTTAGGAGCTAGAAGGAGCCT		
Alu (c19J) [h]	F	CTACTCAAAATATTAAACATAGGC	53 °C	25-30
	R	GCTGCAACGCTGCTATGAAC		
Alu (c22) [h]	F	GTTCTGACACACTTGGAGAAA	53 °C	25-30
	R	GTTGTTGTTATTGCACAACTCA		
Alu (c22) downstream[c] [h]	F	CCTCAGACCCTCAAAATTGC	58°C	25-30
	R	ACGCACTGACCACATGAGAG		
Alu (MLL) [m]	F	CGATTACCCTGTTATCCCTAGGCTGGGCACAGTGGT	60 °C	25-30
	R	AAGCTAGCGGCTGAAATTCTCCTCTTC		
APC [h]	F	GAGGAAGGTGAAGCACTCAGTT	60 °C	23-25
	R	AGGGTGAGACATGGAGAGAAGA		
Apo-E [m]	F	TTCGGAAGGAGCTGGTAAGAC	57 °C	23-25
	R	CGACAGTCCCGTACTCCTTC		
Apo-E [h]	F	CAGCGGAGGTGAAGGACGTC	57 °C	23-25
	R	CTCCTCCTCTCCCCAAG		
Apo-E mRNA [m]	F	GTTTCGGAAGGAGCTGACTG	57 °C	30-32
	R	AGCGCAGGTAATCCCAGAAG		
Apo-E mRNA [h]	F	GGTCGCTTTTGGATTACCT	57 °C	30-32
	R	TTCCTCCAGTTCCGATTTGT		
ARPP P0 [h,m]	F	GCACTGGAAGTCCAACTACTTC	58 °C	16-20
	R	TGAGGTCCTCCTTGGTGAACAC		
B1 consensus[d] [m]	F	TGGTGGTGCATGCCTTTAAT	58 °C	10-12
	R	CCTGGTGTCCTGGAACTCACT		
B2 consensus[e] [m]	F	GGGGCTGGAGAGATGGCT	58 °C	10-12
	R	CCATGTGGTTGCTGGGAT		
B1 (c9) [m]	F	GCATGCATACCACTCCACAC	58 °C	25-30
	R	CAGAGAATCTGCAGTCGTATTTCC		
B2 (c9) [m]	F	CTGCCTTCAGACACACCAGAAG	58 °C	25-30
	R	GATGGAAGAGGTTTTGCCAAG		
c-Myc mRNA [h]	F	TCTGAGGAGGAACAAGAA	58 °C	25-30
	R	GAAGGTGATCCAGACTCT		
GAPDH mRNA [m]	F	TCCACCACCCTGTTGCTGTA	60 °C	23-25
	R	ACCACAGTCCATGCCATCAC		
p53BP2 mRNA [m]	F	GTTGGTTTCGGCGAGAAGG	60 °C	23-25
	R	GAAGCCAAGCGAGAACGAG		
p21 promoter [m]	F	CTCTGGGAAGCCAGAAGTTGTT	58 °C	25-30
	R	GGTCCAGTCCTGCATCTAAGT		
p21 promoter [h]	F	TATTGTGGGGCTTTTCTG	58 °C	25-30
	R	CTGTTAGAATGAGCCCCCTTT		
pre-tRNA^{Leu} [h,m]	F	GTCAGGATGGCCGAGTGGTCTAAGGCGCC	68 °C	20-25
	R	CCACGCCTCCATACGGAGACCAGAAGACCC		
pre-tRNA^{Tyr} [h,m]	F	CCTTCGATAGCTCAGCTGGTAGAGCGGAGG	65 °C	20-25
	R	CGGAATTGAACCAGCGACCTAAGGATCTCC		

SUPPLEMENTARY REFERENCES

1. Hellmann-Blumberg U, Hintz MF, Gatewood JM, Schmid CW. Developmental differences in methylation of human Alu repeats. *Molecular and cellular biology* **13**, 4523-4530 (1993).
2. Liu WM, Maraia RJ, Rubin CM, Schmid CW. Alu transcripts: cytoplasmic localisation and regulation by DNA methylation. *Nucleic acids research* **22**, 1087-1095 (1994).
3. Allen TA, Von Kaenel S, Goodrich JA, Kugel JF. The SINE-encoded mouse B2 RNA represses mRNA transcription in response to heat shock. *Nature structural & molecular biology* **11**, 816-821 (2004).
4. den Dunnen JT, Schoenmakers JG. Consensus sequences of the Rattus norvegicus B1- and B2 repeats. *Nucleic acids research* **15**, 2772 (1987).
